# Effect of alkali metal cations on dehydrogenative coupling of formate anions to oxalate

**DOI:** 10.3389/fchem.2025.1588773

**Published:** 2025-04-23

**Authors:** Atsushi Tahara, Aska Mori, Jun-ichiro Hayashi, Shinji Kudo

**Affiliations:** ^1^ Creative Interdisciplinary Research Division, Frontier Research Institute for Interdisciplinary Sciences (FRIS), Tohoku University, Sendai, Japan; ^2^ Graduate School of Pharmaceutical Sciences, Tohoku University, Sendai, Japan; ^3^ Division of Advanced Device Materials, Institute for Materials Chemistry and Engineering (IMCE), Kyushu University, Fukuoka, Japan

**Keywords:** formate anion, oxalate dianion, CO_2_, alkali metal cations, DFT calculation

## Abstract

**Introduction:**

With the growing global concern over CO_2_ emissions, reducing CO_2_ output has become an urgent requirement. The iron production industry is among those with the highest CO_2_ emissions, primarily due to the use of coke as a reductant and the use of a heat source at approximately 2,000°C. To address this issue, various alternative reductants, including CO, H_2_, and lignite, have been explored. Building on these efforts, we recently reported a novel ironmaking system using oxalic acid (HOOC–COOH) as the reductant. Formate salts, hydrogenated forms of CO_2_, are promising precursors for oxalate salts; however, their behavior during dimerization remains poorly understood. Herein, we investigate the influence of group 1 and 2 metal cations on the base-promoted dehydrogenative coupling of formate to form oxalate.

**Methods:**

First, dehydrogenative coupling of sodium formate was executed by using various types of groups 1 and 2 metal carbonates. Second, the base was replaced from metal carbonates to metal hydroxides to check the reactivity. Finally, a countercation of sodium formate was replaced to various types of groups 1 and 2 metals. To elucidate the reaction mechanism, DFT calculation was executed.

**Results and discussion:**

Treatment of sodium formate with various bases (group 1 and 2 metal carbonates or hydroxides) revealed that group 1 metal hydroxides are more effective than metal carbonates for oxalate formation, with cesium hydroxide (CsOH) exhibiting high reactivity. Density functional theory (DFT) calculations suggest that this kinetic advantage arises not only from increased basicity but also from intermediate destabilization in the Na/Cs mixed-cation system. Additionally, both experimental and theoretical investigations reveal that oxalate yield is influenced by the thermodynamic stability of intermediates and products (oxalate salts), highlighting the crucial role of cations in the reaction.

## 1 Introduction

Oxalic acid (HOOC–COOH) is the simplest dicarboxylic acid (Riemenschneider and Tanifuji, 2011; Schuler et al., 2021a). It undergoes thermal decomposition to form CO_2_ and formic acid, and formic acid further decomposes into water and CO in the presence of an acid. Its conjugate base, the oxalate dianion, is a reducing agent that acts as a two-electron donor, which subsequently decomposes into two CO_2_ molecules (Gibson et al., 2016). Oxalic acid is found in many plants, and its excessive ingestion and prolonged exposure to the skin are potential safety concerns (Zuo et al., 2016). Oxalic acid is widely used as a chemical feedstock in various industries including dyeing (Lee et al., 1999) and as an extractant in metallurgy of lanthanides from its ores (Abrahama et al., 2014; Zhang et al., 2022). In contrast to these conventional uses, our research team has proposed a novel ironmaking process with oxalic acid as the reductant (Santawaja et al., 2020; Santawaja et al., 2022). In iron and steel industries, high CO_2_ emission is one of the most pressing problems against sustainability (IEA. 2020). Traditionally, iron (0) is produced by the reaction of iron oxide with coke [C(0)] to produce CO_2_ as a byproduct. In the method proposed by our team, oxalic acid is used as a reductant instead of coke. The reaction of iron oxide with oxalic acid results in the formation of iron (III) oxalate, which was photochemically and thermally reduced to iron (0) powder concomitantly with the degradation of oxalate dianion to CO_2_ (Parker and Hatchard, 1959; Dudeney and Tarasova, 1998; Ogi et al., 2015). The regeneration of oxalic acid from CO_2_ could lead to the development of a sustainable carbon-neutral ironmaking system.

**FIGURE 1:**
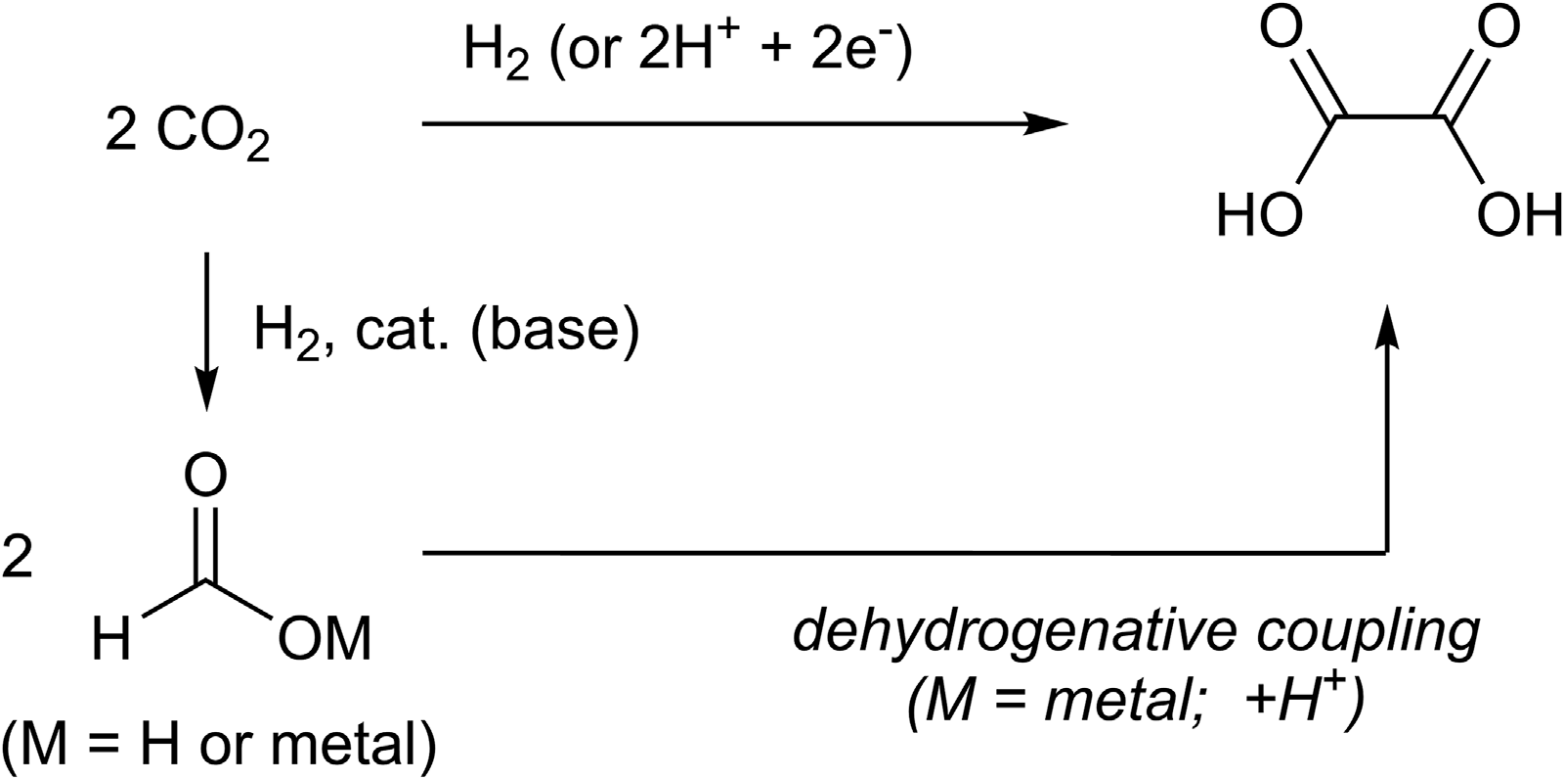
Syntheses of oxalic acid from CO_2_, formic acid, or metal formats.

Oxalic acid has been traditionally produced from non-CO_2_ carbon sources (Schuler et al., 2021b; Fenton and Steinwand, 1974). Meanwhile, the reductive coupling of CO_2_ to form oxalate dianions has been examined in electrochemical studies (Savéant, 2008) and extended to the fields of metal-complex-catalyzed electrochemistry (Becker et al., 1985; Kushi et al., 1994; Kushi et al., 1995; Evans et al., 1998; Angamuthu et al., 2010) and biochemistry (Pastero et al., 2019) (Figure 1). Kanan et al. reported that thermal coupling of CO_2_ to form > C_2_ fragments, including oxalate dianions, using alkali metal carbonates is a non-electrochemical or non-biochemical approach (Banerjee and Kanan, 2018). In this approach, formate anions were produced first, followed by carbonite (CO_2_^2–^) species. This approach not only produced oxalic acid but also promoted C_2_ (and >C_2_) chemistry via the transformation of oxalate to other organic compounds such as glycolic acid (Schuler et al., 2021a; Watanabe et al., 2015).

There have been limited reports on the production of >C_2_ compounds, apart from oxalic acid, that use CO_2_ as a direct feedstock (Banerjee and Kanan, 2018; Prieto, 2017). In contrast, C_1_ compounds produced via CO_2_ reduction have been studied extensively, some of which find industrial applications (Álvarez al., 2018). Therefore, it is reasonable to synthesize oxalic acid by coupling formic acid or its salt that are potentially available via the reduction of CO_2_. To form a carbon–carbon bond from two formate anions, one formyl H atom, which normally acts as a *δ* ^–^H atom or hydride (H^−^), must be abstracted as a proton (H^+^). The resulting carbonite species (CO_2_^2–^) binds to the other formyl anion to form the oxalate species following hydrogen abstraction from the hydridic C–H bond. This reaction mechanism has been proposed in several reports (Andresen, 1977; Górski and Kraśnicka, 1987; Górski and Kraśnicka, 1987). The reaction is carried out in the presence of a base, typically an alkali carbonate. However, excess CO_2_ is generated when carbonate salts are used. Other reagents such as sodium amide or sodium borohydride were also used. As a breakthrough, in 2016, Lakkaraju and Batista et al. reported the dehydrogenative coupling of sodium formate to obtain the oxalate salt in the presence of a catalytic amount of sodium hydride (NaH) (Lakkaraju et al., 2016). Schuler et al. extended their approach to various types of bases, and it was found that potassium hydride (KH) and lithium hydride (LiH) also showed high catalytic activity at lower reaction temperatures, similar to NaH (Schuler et al., 2021a; Schuler et al., 2021b).

Given their report, we focused on the behavior of cations in the feedstock formate and bases during the coupling reaction. In the report by Lakkaraju and Batista et al., the catalytic activities of sodium hydroxide (NaOH) and potassium hydroxide (KOH) were investigated. Their activity was lower than that of NaH. It should be noted that not only the difference in anions (*i.e.*, hydroxide and hydride) but also the difference in metal cations (*i.e.*, Na^+^ and K^+^) affected the oxalate yield (Lakkaraju et al., 2016). In the report by Schuler, KH showed the highest catalytic activity compared with those of NaH or LiH (Schuler et al., 2021a; Schuler et al., 2021b). Although the reactivity of the other alkali metals (*i.e.*, Rb^+^ and Cs^+^) seems to have generated interest, only cesium carbonate was studied in the report. Moreover, the effects of several types of metal cation combinations on the substrate (formate), product (oxalates), and bases have not been investigated in parallel, although self-thermolysis of formate salts bearing group 1 and 2 metal cations has been reported (Górski and Kraśnicka, 1987; Shishido and Masuda, 1971).

Herein, we report the base-promoted dehydrogenative coupling of the formate salts bearing different metal cations to oxalates. First, the dehydrogenative coupling of sodium formate was performed using various metal carbonates or hydroxides as bases. Based on the screening, it was observed that heavier alkali hydroxides showed higher reactivity toward the dimerization. Using the information obtained from density functional theory (DFT) calculations, the theoretical explanation for the reaction mechanism, in particular, the role of metal cations has been elucidated. The Na^+^ in the formate salts were also replaced with the other group 1 and 2 metal cations and evaluated in a similar manner.

## 2 Materials and methods

### 2.1 Experimental section

#### 2.1.1 General procedure

Groups 1 and 2 metal formates, metal carbonates, metal hydroxides, and conc. HCl_(aq)_ were purchased from Wako Pure Chemical Industries, Nacalai Tesque, Kanto Chemical, or Sigma-Aldrich. DSC measurements were performed using NETZSCH DSC 204 F1 Phoenix. Details of the reactor used for the coupling reaction are described in Supplementary Figure S1. The yield of oxalic acid was determined by HPLC analysis on a SHIMADZU HPLC unit (Prominent series), including the following instruments: a pump, a PDA detector, a column oven, and the Bio-Rad Aminex 87H column.

#### 2.1.2 DSC measurement

Metal formate (5 mg) and metal carbonate or metal hydroxide (0.3 equivalents per mol) were set on a platinum pan. Under a flow of N_2_ gas (100 mL/min), DSC measurement for the mixture was performed at a temperature range of 30°C to 460 °C at a heating rate of 5 or 30°C/min. All the results are summarized in Supplementary Figures 2–5.

#### 2.1.3 Synthesis of oxalic acid from metal formates

Metal formate (45.0 mmol) and the base (metal carbonate or metal hydroxide, 15.0 mmol) were added to a 15-mm *ϕ* glass insert and set to the reactor with a thermometer (drawn details are in Supplementary Figure S1). Under a flow of N_2_ gas (300 mL/min), the mixture was heated at 360°C for 1 h. After cooling, the resulting solid was dissolved in H_2_O, and conc. HCl_(aq)_ was added until pH = 1. The water was removed under reduced pressure, and the white solid obtained was analyzed by HPLC to determine the yield of oxalic acid (mobile phase; 5 mM H_2_SO_4_, flow rate; 0.6 mL/min, R_t_ = 7.2 min, λ = 210 nm for oxalic acid.).

### 2.2 Computational methods

#### 2.2.1 DFT calculations

All calculations were performed using the Gaussian 16 rev. C program to search for all intermediates and transition structures on potential energy surfaces (Frisch et al., 2016). For optimization, the B3LYP-D functional was selected (Frisch et al., 2016; Grimme, 2006). We also employed the SDD (Stuttgart/Dresden pseudopotentials) (Andrae et al., 1990) and 6-31G** basis sets (Gordon, 1980; Hariharan and Pople, 1974; Hariharan and Pople, 1973; Hehre et al., 1972; Ditchfield et al., 1971) for group 1 metal atoms (Li, Na, K, Rb, and Cs) and the other atoms, respectively [BS1]. All stationary-point structures were found to have an appropriate number of imaginary frequencies. An appropriate connection between a reactant and a product was confirmed by the intrinsic reaction coordinate (IRC) (Fukui, 1970; Fukui, 1981; Gonzalez and Schlegel, 1990) and quasi-IRC (qIRC) calculations. In the quasi-IRC calculation, the geometry of a transition state was first shifted by perturbing the geometries very slightly along the reaction coordinate and then released for equilibrium optimization. To determine the energy profile of the proposed reaction scheme, we performed single-point energy calculations at the optimized geometries using the SDD (Stuttgart/Dresden pseudopotentials) and 6-311++G** (Krishnan et al., 1980; McLean and Chandler, 1980; Clark et al., 1983) basis sets for group 1 metal atoms (Li, Na, K, Rb, and Cs) and the other atoms, respectively. The solvent effects of the molten salt (*ε* = 26.0) were evaluated using the polarizable continuum model (PCM) (Miertuš et al., 1981) [BS2]. Energy profiles of the calculated reaction pathways are presented as Gibbs free energy changes (Δ*G*) involving thermal corrections at 298.15 K. All the optimized structures (ball-and-stick models) and optimized geometries in the XYZ file format are summarized in Supplementary Tables 1–11.

## 3 Results

We report the formation of sodium oxalate via thermolysis of sodium formate at 360 °C in the presence of Na_2_CO_3,_ followed by quenching with conc. HCl_(aq)_. However, 2.0 equivalents of the base was required to quantitatively obtain oxalic acid (Andersen, 1977), and the base was decomposed into CO_2_ during quenching. Therefore, the relationship between the amount of Na_2_CO_3_ and oxalate yield was investigated before screening for the base. As shown in Table 1, quantitative formation of oxalic acid is observed when more than 0.5 equivalents of Na_2_CO_3_ (entries 1–4) is used, which are not helpful to compare the effects of the base. When the amount of the base was reduced to 0.3 equivalents, the yield decreased to 59% (entry 5), in which the moderate yield at the initial reaction condition enables both improved and less efficient outcomes to be observed by changing the bases. The oxalic acid could not be obtained in the absence of a base at 360 °C but at 410 °C (entries 6,7), which is in good correspondence with a previous report about self-dimerization of sodium formate without bases at the range 390°C–400 °C (Schuler et al., 2021a). The relatively low yield of oxalic acid (40%) is possibly due to the competitive reaction of its thermal degradation. No oxalic acid was produced in the absence of sodium formate (entry 8).

**TABLE 1:** Reaction of sodium formate with different equivalents of sodium carbonate^a^.

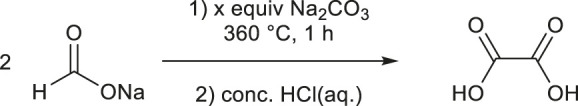		
Entry	Equivalent of Na_2_CO_3_	Yield [%]^b^
1	2.0	96
2	1.5	97
3	1.0	98
4	0.5	91
5	0.3	59
6	0.0	0
7^c^	0.0	40
8^d^	[45.0 mmol]	0

^a^ All reactions were carried out using sodium formate (3.06 g, 45.0 mmol), Na_2_CO_3_ (0.0–90.0 mmol, 0.0–2.0 equivalent toward sodium formate), at 360 °C for 1 h, followed by quenching with conc. HCl_(aq)_.

^b^ Determined using HPLC, the average yield of the three measurements is listed, of which the standard deviation is below 2.5%.

^c^ 410 °C.

^d^ 0.0 mmol sodium formate was used.

To evaluate the appropriate combination of base and metal formate, the amount of base for the coupling of metal formate was fixed at 0.3 equivalents. Before starting the experiments, differential scanning calorimetric (DSC) measurements of these mixtures were obtained. Some combinations showed large peaks at approximately 300°C–400 °C, which confirmed the exothermic coupling reaction (see Supplementary Figure S2). Table 2 shows the coupling of sodium formate with 0.3 equivalents of metal carbonates (group 1 and 2 metals). Li_2_CO_3_ weakly promoted the dehydrogenative coupling of sodium formate and gave a 14% yield of oxalic acid (entry 1). The other group 1 carbonate salts (*i.e.*, K_2_CO_3_, Rb_2_CO_3_, and Cs_2_CO_3_) showed no reactivity (entries 3–5). Although MgCO_3_ and CaCO_3_ were inactive, some group 2 carbonate salts, such as SrCO_3_ and BaCO_3_, showed mild reactivity, resulting in a 40% and 50% yield of oxalic acid, respectively (entries 6–8, 11). An increase in the reaction temperature with use of SrCO_3_ and BaCO_3_ as bases reduced the yield, which was due to the thermal degradation of the generated oxalate salts (entries 8–13) (Górski and Kraśnicka, 1987; Shishido and Masuda, 1971). A unique reactivity was observed in the strontium and barium salts; however, the yields were less than those with Na_2_CO_3_.

**TABLE 2:** Reaction of sodium formate with different group 1 and 2 metal carbonates.^a^.

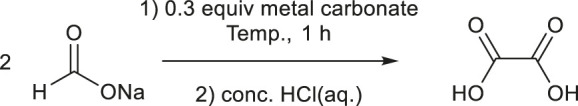			
Entry	Base	Temp. [°C]	Yield [%]^b^
1	Li_2_CO_3_	360	14
2^c^	Na_2_CO_3_	360	59
3	K_2_CO_3_	360	<1
4	Rb_2_CO_3_	360	<1
5	Cs_2_CO_3_	360	<1
6	MgCO_3_	360	2
7	CaCO_3_	360	<1
8	SrCO_3_	360	40
9	SrCO_3_	380	34
10	SrCO_3_	410	28
11	BaCO_3_	360	50
12	BaCO_3_	380	37
13	BaCO_3_	410	35

^a^ All reactions were carried out using sodium formate (3.06 g, 45.0 mmol) and metal carbonate (15.0 mmol) for 1 h, followed by quenching with conc. HCl_(aq)_.

^b^ Determined using HPLC, the average yield of the three measurements is listed, of which the standard deviation is below 2.5%.

^c^ The same data as in Table 1.

Next, the reactivities of group 1 and 2 metal hydroxide salts were investigated. The results are shown in Table 3. When NaOH was used as a base, 71% oxalic acid yield was obtained, which was higher than that obtained using Na_2_CO_3_ (entry 2). Not only NaOH but all the group 1 metal hydroxides also showed moderate or good reactivities (entries 3–5). Among group 2 metal hydroxides, however, only Mg(OH)_2_ showed good reactivity (entry 6), although it cannot be ignored that 0.6 equivalents of hydroxyl anions was generated from 0.3 equivalents of Mg(OH)_2_, in which additional –OH may affect the outcome.

**TABLE 3:** Reaction of sodium formate with different group 1 and 2 metal hydroxides.^a^.

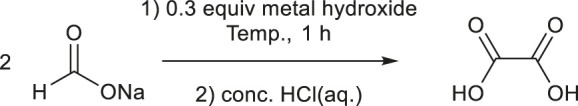			
Entry	Base	Temp. [°C]	Yield [%]^b^
1	LiOH	360	43
2	NaOH	360	71
3	KOH	360	63
4	RbOH	360	72
5	CsOH	360	74
6	Mg(OH)_2_	360	69
7	Ca(OH)_2_	360	<1
8	Sr(OH)_2_	360	7
9	Ba(OH)_2_	380	17
10	NaOH	350	68
11	NaOH	340	70
12	NaOH	320	72
13	NaOH	300	73
14	NaOH	280	44
15	NaOH	250	2
16	CsOH	340	82

^a^ All reactions were carried out using sodium formate (3.06 g, 45.0 mmol) and metal hydroxide (15.0 mmol) for 1 h, followed by quenching with conc. HCl_(aq)_.

^b^ Determined using HPLC, the average yield of the three measurements is listed, of which the standard deviation is below 2.5%.

Further analysis revealed that lowering the reaction temperature improved the oxalic acid yield. In the present study, the reaction at 340 °C using CsOH gave the highest yield of oxalic acid (82%, entry 16). Using NaOH, oxalic acid was obtained at the lowest reaction temperature, 280 °C, without a decrease in the yield (entries 10–15). These trends could be rationalized by the thermal degradation of the resulting oxalate anions at higher temperatures (Górski and Kraśnicka, 1987; Shishido and Masuda, 1971).

Based on Tables 2 and 3, there is potential that the use of similar cations could affect the reactivity. A screening for formate salt cations was then performed. However, as summarized in Table 4, except for the combination of potassium formate and KOH, which had a 40% yield (entry 6), most metal formates could not be dimerized in the presence of carbonate or hydroxide salts having the same metal cations. These results indicate that similar cations do not always promote homocoupling. Entries 13 and 14 show possible cation exchange between formate salts and bases. When cesium formate was treated with NaOH, 65% oxalic acid was obtained, which was similar to that obtained from the combination of sodium formate and CsOH, as shown in Table 3.

**TABLE 4:** Reaction of metal formates with metal hydroxides.^a^.

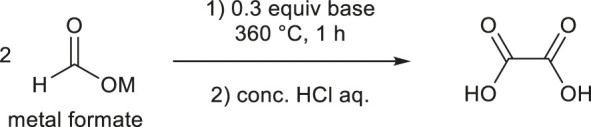			
Entry	Formate	Base	Yield [%]^b^
1	Li [O_2_CH]	Li_2_CO_3_	<1
2	Li [O_2_CH]	LiOH	<1
3^c^	Na [O_2_CH]	Na_2_CO_3_	59
4^c^	Na [O_2_CH]	NaOH	71
5	K [O_2_CH]	K_2_CO_3_	<1
6	K [O_2_CH]	KOH	40
7^d^	Cs [O_2_CH]	Cs_2_CO_3_	6
8	Cs [O_2_CH]	CsOH	<1
9	Mg [O_2_CH]_2_	MgCO_3_	<1
10	Mg [O_2_CH]_2_	Mg(OH)_2_	<1
11	Ca [O_2_CH]_2_	CaCO_3_	<1
12	Ca [O_2_CH]_2_	Ca(OH)_2_	<1
13^e^	Cs [O_2_CH]	Na_2_CO_3_	8
14	Cs [O_2_CH]	NaOH	65

^a^ All reactions were carried out using metal formate (45.0 mmol) and metal hydroxide (15.0 mmol) at 360 °C for 1 h, followed by quenching with conc. HCl_(aq)_.

^b^ Determined using HPLC, the average yield of the three measurements is listed, of which the standard deviation is below 2.5%.

^c^ Same data as in Tables 1–3.

^d^ 430°C.

^e^ 400 °C.

## 4 Discussion

The experimental results of base-promoted dehydrogenative couplings to form oxalate anions are summarized as follows: (1) sodium cations are appropriate when using carbonate salts as the base. Strontium and barium were relatively efficacious. (2) All group 1 metal hydroxides were better bases than metal carbonates, of which CsOH showed the highest reactivity. With the use of highly reactive bases such as NaOH and CsOH, the reaction temperature can be lowered down to 280 °C, enabling suppression of the thermal decomposition of the products. (3) The unification of metal cations between the formate salts and bases was not effective to improve the yield of oxalic acid. (4) A cation exchange between the formate salts and bases may occur during the reaction.

One of the biggest differences between metal carbonates/hydroxides/hydrides is the basicity. The strength of the basicity increases in the order of CO_3_^2–^ < OH^−^ < H^−^ (Lew, 2009), which explains the high yield of oxalic acid when using MOHs than when using carbonate salts. Although the result using CsOH could not reach those observed in the previous report using NaH in both catalytic amount (2 wt%, ca. 5.7 mol% toward sodium formate) and yield (99%) (Lakkaraju et al., 2016), or the combination of potassium formate and NaH or KH in the reaction temperature (below 200 °C) (Schuler et al., 2021b), metal hydroxides could be chosen as efficient bases for dimerization without further care of air (no need to use a glovebox). Notably, metal hydroxides also show a hygroscopic property, so usage of a glovebox may improve our results further.

It should be noted that not only the kind of anions but cations also affect the yield of oxalate salts. In addition to the results reported previously, with use of NaOH and KOH (Lakkaraju et al., 2016; Schuler et al., 2021a; Schuler et al., 2021b), the reactivities of LiOH, RbOH, and CsOH were reinvestigated, and it was revealed that heavier bases also worked as a good base for oxalate formation. It is well known that alkali metal hydroxides dissociate completely in solution to form M^+^ and OH^−^, and the basicity of group 1 metal hydroxides is nearly similar in a dilute solution. However, in a concentrated solution or a molten salt state, the strength of the basicity increases in the order of LiOH < NaOH < KOH < RbOH < CsOH due to the increase in the ionic radius of metal cations (Kennedy, 1938).

To acquire mechanistic insights and to compare the activation energy barriers between several bases, a computational study was performed. To simplify the discussion, the reactions using group 1 metal hydroxides were selected for the study. The group 1 metal carbonate salts or group 2 metal hydroxides/carbonates were not considered for the computational study because multivalent ions (CO_3_^2–^ or M^2+^) have the potential for multiple coordination styles to generate several reaction routes (see below).

**FIGURE 2:**
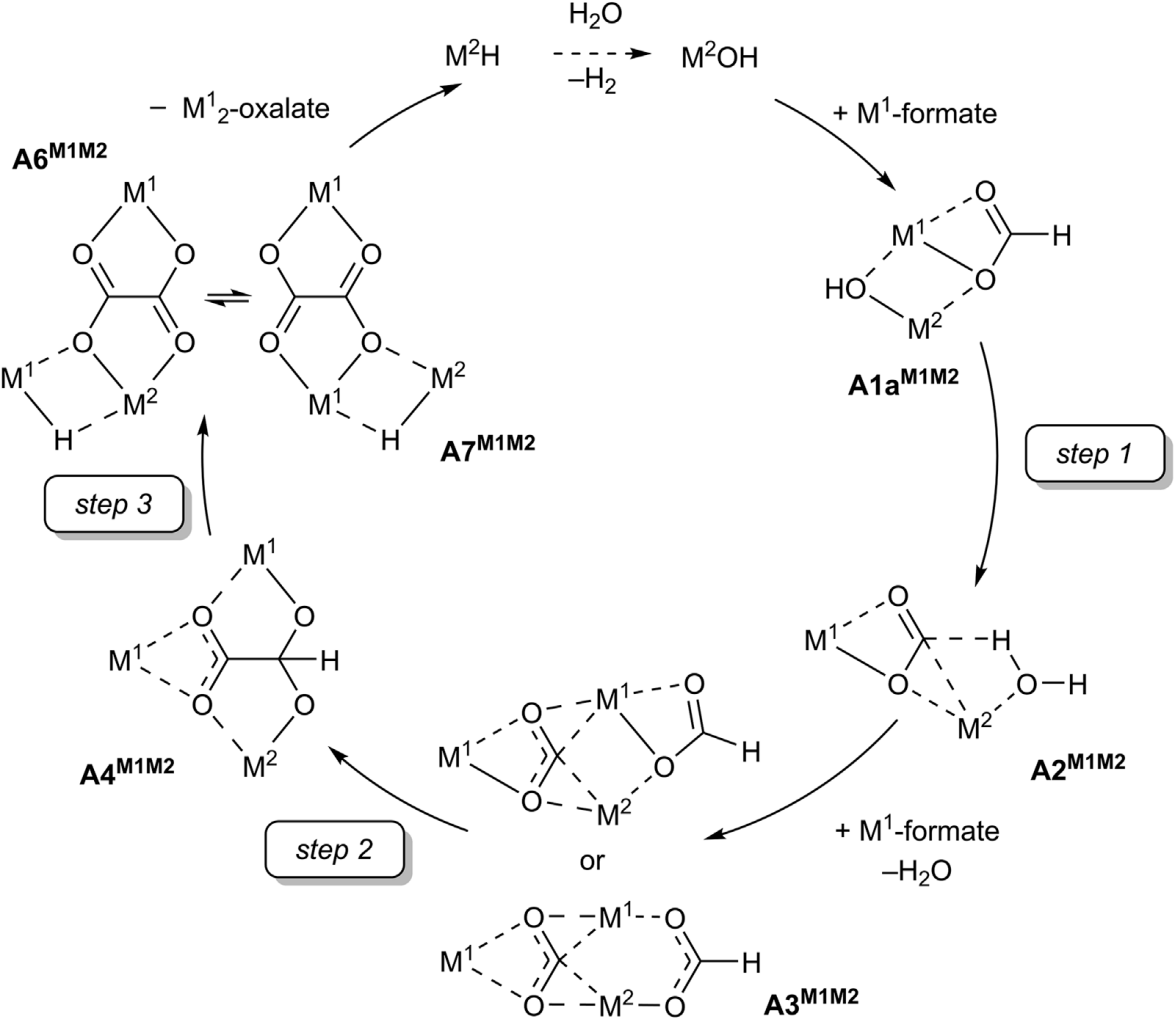
Proposed reaction scheme for the dehydrogenative dimerization of formate anions to oxalate (Lakkaraju et al., 2016).

Lakkaraju and Batista et al. proposed a reaction mechanism for the metal hydride-catalyzed dehydrogenative coupling of sodium formate (Lakkaraju et al., 2016), which consists of three main steps: (*step 1*) deprotonation of the formyl proton of the formate anion to form carbonite species, (*step 2*) C–C bond formation between (formally) dianionic carbonite and formate species stabilized by metal cations to form hydrooxalate species, and (*step 3*) dehydridation of hydrooxalate trianion to form metal hydride and oxalate species. In the previous study that used NaH as the catalyst, H_2_ was generated as a by-product in *step 1* (Lakkaraju et al., 2016). In the present work, H_2_O is assumed to be obtained from metal hydroxide in *step 1*. Water then reacted with the metal hydride formed in *step 3* to concomitantly regenerate the metal hydroxide and produce H_2_. In the absence of water, MOH may promote the reaction quantitatively, not catalytically, and the resulting MH could behave as a catalyst. In the gaseous outlet stream, detection of water was observed during the reaction by using GC, so there seems to be a low possibility of the regeneration of MOH species. On the other hand, if the 0.3 equivalent of MOH was fully consumed as the base, 60% of oxalate salt could be theoretically formed, while up to 82% of oxalic acid was experimentally obtained. Therefore, we cannot ignore three possibilities (case 1, MOH as stoichiometric; case 2, MOH as catalytic; and case 3, MOH as the first cycle and then MH after the second cycle). In this paper, the pathway using MOH was calculated to consider the complementarity with Lakkaraju and Batista’s report (Lakkaraju et al., 2016). The overall reaction scheme in the case of MOH is shown in Figure 2.

The reactions shown in Table 3, *i.e*.*,* dehydrogenative coupling of formate anions assisted by group 1 metal hydroxides (LiOH, NaOH, KOH, RbOH, and CsOH), were calculated, some of which have already been reported (Lakkaraju et al., 2016). All calculation data are given in Supplementary Tables 1–11.

The total Gibbs free energy of the free formate salt and metal hydroxide was set to 0 kcal/mol. The mixture of sodium formate and metal hydroxide was stabilized by aggregation to form **A1^NaM2^**. As shown in Table 5, there are three types of isomers for **A1^NaM2^**: *κ* ^2^-formate anion on the Na atom (**A1a^NaM2^**), *κ* ^2^-formate anion on the M^2^ atom (**A1c^NaM2^**), and *µ*-*κ* ^1^:*κ* ^1^-formate on both Na and M^2^ atoms (**A1b^NaM2^**) with bridged hydroxide anions. Each isomer was formed exergonically from the free formate salt and a metal hydroxide. When M^2^ = Na, **A1a^NaNa^** and **A1c^NaNa^** were same, and the Gibbs free energy (−4.1 kcal/mol) was higher than that of the bridging formate, **A1b^NaNa^** (−5.9 kcal/mol). However, the heavier cations, namely, K, Rb, and Cs, did not produce *µ*-*κ* ^1^:*κ* ^1^-formate species as optimized structures to converge into **A1c^NaM2^**, which was not energetically preferred compared to the normal adduct, **A1a^NaM2^**. This may be due to the instability that occurs because of the difference between two cations with different atomic radii. With sodium formate and LiOH, only **A1b^NaLi^** was formed, and it exhibited lower relative Gibbs free energy (−12.1 kcal/mol).

**TABLE 5:** Relative Gibbs free energy (ΔG [kcal/mol]) for the adduct of sodium formate with various metal hydroxides^a^.

M^2^	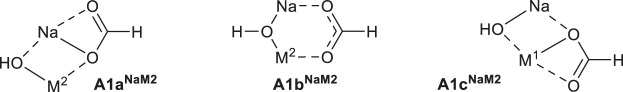		
Li	Conv. to **A1c^NaLi^**	−12.1	Conv. to **A1c^NaLi^**
Na	−4.1	−5.9	Same as **A1a^NaNa^**
K	−3.2	Conv. to **A1c^NaK^**	−3.0
Rb	−4.9	Conv. to **A1c^NaRb^**	−4.3
Cs	−5.2	Conv. to **A1c^NaCs^**	−3.8

^a^ For optimization: B3LYP level using SDD (for Li, Na, K, Rb, and Cs) and 6-31G** (C, H, and O) basis set. For energy calculations, B3LYP-D level using SDD (for Li, Na, K, Rb, and Cs) and 6-311++G** (C, H, and O) basis set, including the solvent effect (PCM, *ε* = 26.0 for molten salt).

Next, the activation energy barrier for *step 1* was estimated computationally. Since **A1^NaM2^** was just a resting state, M^2^OH was needed to move next to the H atom of the formate salt. Deprotonation (*note: not dehydridation*) of the formic H atom by the hydroxyl group occurred via the transition state TS_A1/A2_^NaM2^ (see detailed structures in Supplementary Table 2), and the resulting carbonite species was stabilized with the assistance of M^2^ to form **A2^NaM2^**. Table 6 shows the activation energy barriers for *step 1* (energy gap between **TS_A1/A2_^NaM2^** and **A1^NaM2^**) for different metal hydroxides. Compared with NaOH (Δ*G* ^‡^_step1_ = 36.6 kcal/mol), those for KOH, RbOH, and CsOH exhibited relatively small Δ*G* ^‡^_step1_ (ca 30 kcal/mol). This behavior could be attributed to not only the stabilization of transition states due to the increase in the basicity but also the destabilization of the starting species, A1^NaM2^, as explained above. The thermodynamic stabilities of the resulting carbonite species are similar (Δ*G* = 33.3–34.5 kcal/mol, see Supplementary Table 1 in SI) regardless of the metal hydroxide used. For LiOH, the structure of the corresponding transition state TS_A1/A2_^NaLi^ could not converge either due to its weak basicity or the strong affinity between Li and O (see below). The relative Gibbs energies of the carbonite species (M^1^ = Na, M^2^ = Li) were estimated to be 29.9 kcal/mol by the comparison of Δ*G* (sodium formate and LiOH) and Δ*G* (sodium lithium carbonite and water), as shown in Supplementary Table 2.

**TABLE 6:** Activation energy barriers (ΔG^‡^ [kcal/mol]) for *steps 1–3* described in Figure 2.^a^

M^1^	M^2^	Step 1	Step 2	Step 3
Na	Li	Not converged^b^	26.2	8.4
Na	Na	36.6^b^	25.7	14.3
Na	K	30.6^c^	23.8	14.9
Na	Rb	29.8^c^	23.9	15.4
Na	Cs	29.8^c^	23.5	15.7
Li	Na	40.5^b^	28.5	13.6

a For optimization: B3LYP level using SDD (for Li, Na, K, Rb, and Cs) and 6-31G** (C, H, and O) basis set. For energy calculations, B3LYP-D level using SDD (for Li, Na, K, Rb, and Cs) and 6-311++G** (C, H, and O) basis set, including the solvent effect (PCM, ε = 26.0 for molten salt).

b A1bNaM2 to TS_A1/A2_NaM2.

c from **A1aNaM2** to TS_A1/A2_NaM2.

The resulting H_2_O molecule in **A2^NaM2^** is replaced with the other sodium formate to form **A3^NaM2^**. In **A3^NaM2^**, a C–C bond is formed to generate **A4^NaM2^** (*step 2*). Although the energy barrier for *step 2* exhibits a marginal trend (Li > Na > K > Rb, Cs), as shown in Table 6, these differences are relatively small compared to those for *step 1*. **A4^NaM2^** bears a hydrooxalate trianion that interacts with two Na cations and one M^2^ cation. The dehydridation of hydrooxalate H atom by the M^2^ cation produced an adduct, **A5^NaM2^**, composed of oxalate species and a metal hydride species (*step 3*). The remaining hydride is located between the M^2^ atom and the Na atom bearing one carboxylate group. Unlike for *steps 1* and *2*, Δ*G* ^‡^ for *step 3* increases in the order Li < Na < K < Rb < Cs due to the increase in the stability of **A4^NaM2^** bearing heavier cations (right column in Table 6). The energy differences for *step 3* for all M^2^ cations were lower.

There are two possible oxalate salt/metal hydride combinations. One is sodium oxalate, where both cations are Na atoms, and M^2^ hydride (**A7^NaM2^**). The other is an oxalate salt containing one Na and M^2^ atom and NaH (**A6^NaM2^**). According to the proposed mechanism, **A6^NaM2^** is first produced and is then isomerized to **A7^NaM2^**. For K, Rb, and Cs, **A7^NaM2^** is thermally stable compared to **A6^NaM2^**, which is reasonable for regenerating M^2^OH by hydration and completing the catalytic cycle (see details in the Supplementary Tables 1–11). Unlike the other four heavy metals of group 1, **A6^NaLi^** is energetically more favorable than **A7^NaLi^**, which results in its degradation into NaOH and [LiNa][oxalate] salt. Although the generation of NaOH seems stoichiometrically unreasonable for the proposed LiOH reaction scheme, these calculations, including a high activation energy barrier for *step 1*, imply that it is NaOH and not LiOH that is the active species, which does not contradict the moderate yield of oxalic acid (entry 1, Table 3).

Furthermore, a computational study was carried out to investigate the effects of alkali cations in formate salts on the coupling reaction. Here, the thermal stability of the resulting metal oxalates depended on the cation of the metal formates (M^1^), whereas the same product, sodium oxalate, was obtained using sodium formate regardless of the base (except for LiOH). A comparison of the Gibbs free energies, as shown in Table 7, reveals that the coupling reactions for K, Rb, and Cs formates are highly endergonic (Δ*G* ≈ 10 kcal/mol) compared to that for sodium (Δ*G* = 5.5 kcal/mol). In addition, most of the intermediates formed via the reaction of K, Rb, and Cs formates were less stable than those formed via Na formate by 5–10 kcal/mol (Table 7). Although the activation energy barriers for *step 1* showed the same tendency as that in Table 3 (exact values are in the Supplementary Tables 1–11), sodium formate gave the best yields due to thermodynamic reasons rather than kinetics.

**TABLE 7:** Relative Gibbs free energy (ΔG [kcal/mol]) for intermediate species described in Figure 2.^a^

M^1^ M^2^	Carbonite + H_2_O	**A3** ^M1M2^	**A6** ^M1M2^	Oxalate + M^2^H	Oxalate + M_2_OH
Li	26.6	18.7	10.9	13.1	−2.1
Na	33.3	30.2	22.6	21.6	5.5
K	38.4	39.4	29.4	25.1	9.6
Rb	39.8	37.8	27.3	24.3	10.3
Cs	43.1	41.7	29.0	25.1	10.9

^a^ For optimization: B3LYP level using SDD (for Li, Na, K, Rb, and Cs) and 6-31G** (C, H, and O) basis set. For energy calculation, B3LYP-D level using SDD (for Li, Na, K, Rb, and Cs) and 6-311++G** (C, H, and O) basis set, including the solvent effect (PCM, ε = 26.0 for molten salt).

Except Li, these results clearly support several points and trends that are listed below: (1) independent of the type of the metal cation, *step 1*, *i.e.*, the generation of the carbonite species, is the rate-determining step, and its activation energy barrier decreases in the order NaOH > KOH > RbOH > CsOH. This supports the experimental results, which indicate that CsOH and RbOH improved oxalic acid yield compared to NaOH. (2) Although *steps 2* and *3* show some trend (Δ*G* ^‡^ decreases for *step 2* and increases for *step 3* from LiOH to CsOH, respectively), the differences are too small to affect the reaction rate. (3) The relative Gibbs free energies for the dimerization of formate salts bearing group 1 metal cations to form their corresponding metal oxalates increased in the order of Na < K < Rb < Cs, which agrees with the experimental results presented in Table 4.

Finally, the discussion will be with regard to the exceptions in the computational trends. For the LiOH/sodium formate combination, the transition state estimation for *step 1* failed. For the NaOH/cesium formate system, all prospective intermediates, including transition states, could not be computationally converged. These failures imply that the proposed configuration has extremely high energy potential to proceed, resulting in the convergence of the other stable structures. However, both mixtures produced oxalic acid in moderate yields (41% and 65%, respectively). These discrepancies between experimental and theoretical results imply that the reaction mechanism depicted in Figure 2 may not describe these two reaction systems and that cation exchange between metal formates and metal hydroxides may occur during the reaction, which easily proceeds via the “bridged” A1b^M1M2^ resting state. The NaOH/cesium formate system behaves as a CsOH/sodium formate system, generating similar amounts of the desired product (74%, as shown in Table 3, entry 5). There are several reports on the cation exchange of Na and Cs in the presence of formate anion to form a complicated crystal structure (Alcock et al., 2006). Although these configurations might be very fluid in molten salt, which does not always form a complicated structure like the crystal, these reports support our proposed hypothesis. We recalculated the reaction route for the NaOH/lithium formate system, assuming cation exchange, and found an activation energy barrier. However, the activation barrier was as high as 40.5 kcal/mol, which implied that reactions without lithium salts (*i.e.,* only the NaOH/sodium formate system worked after Li/Na cation exchange) could not be ignored (Table 6).

As described above, computational studies for only a combination of group 1 metal salts were executed, while those for group 2 were not. Tables 2, 3 suggest that SrCO_3_, BaCO_3_, and Mg(OH)_2_ are efficient bases for the synthesis of oxalate salts. Therefore, it is worth considering their reaction mechanisms. If Mg(OH)_2_ is applied as base to the reaction scheme drawn in Figure 2; thus, the role of an additional hydroxy group in Mg(OH)_2_ needs to be investigated. There are many possibilities of interaction of this hydroxy group, such as whether it activates the same formyl anion, another formyl anion, or stabilizes some intermediates, or remains inert. Furthermore, versatile discussions will be needed when these pathways are extended to SrCO_3_ or BaCO_3_, which consist of multivalent ions (M^2+^ and CO_3_^2–^).

In addition to group 1 and 2 metals, more optimized cation combinations can be employed, including organocations, lanthanides, actinides, and transition metals such as Cu, Zn, Fe, and Al. In particular, one of the ideal goals is to replace group 1 and 2 metal cations in formate salts with iron atoms. If iron formate is dehydrogenatively coupled under similar reaction conditions, the resulting iron oxalate can be utilized directly for the ironmaking system described in the *Introduction*. For such a system, the need to add acid (conc. HCl_(aq)_) for the conversion of oxalate salt into oxalic acid will be eliminated, leading to zero emission of metal waste (MCl, etc.). Concomitant with the development of procedures for the formation of iron formate from CO_2_, H_2_, and iron oxides, a new carbon-cycling ironmaking system could be realized. Studies investigating the thermal degradation of iron formate to iron oxides have been reported (Morando et al., 1987; Viertelhaus et al., 2005). Therefore, a multifaceted discussion is required to determine the most suitable cation for the reaction in terms of stability, reactivity, price, abundance, and safety.

## 5 Conclusion

To summarize, the effect of metal cations during the base-promoted dehydrogenative coupling of formate salts to oxalates was discussed both experimentally and theoretically. Experimentally, it was revealed that metal hydroxides were effective bases for the coupling reaction, compared to carbonate salts, and CsOH showed high activity. Theoretically, DFT calculations suggested that heavier metal hydroxides such as CsOH caused not only stabilization of the transition state (due to stronger basicity) but also destabilization of the initial structure (due to different cations) in the rate-determining step (*i.e.*, the deprotonation of formyl H atom for the formation of carbonite species), leading to the decrease in activation energy barriers. Further attempts to study various cation combinations to realize a carbon-cycling ironmaking system are ongoing.

## Data Availability

The raw data supporting the conclusions of this article will be made available by the authors, without undue reservation.

## References

[B1] Abrahama F. Arab-Chapeletb B. Riveneta M. Tamainb C. Grandjeanb S. (2014). Actinide oxalates, solid state structures and applications. Coord. Chem. Rev. 266-267, 28–68. 10.1016/j.ccr.2013.08.036

[B2] Alcock N. W. Wilson M. P. Rodger P. M. (2006). Caesium sodium bis(formate). Acta Cryst. E 62, m388–m390. 10.1107/S1600536806003047

[B3] Álvarez A. Bansode A. Urakawa A. Bavykina A. V. Wezendonk T. A. Makkee M. (2017). Challenges in the greener production of formates/formic acid, methanol, and DME by heterogeneously catalyzed CO_2_ hydrogenation processes. Chem. Rev. 117, 9804–9838. 10.1021/acs.chemrev.6b00816 28656757 PMC5532695

[B4] Andrae D. Häußermann U. Dolg M. Stoll H. Preuß H. (1990). Energy-adjusted *ab initio* pseudopotentials for the second and third row transition elements. Theor. Chim. Acta 77, 123–141. 10.1007/BF01114537

[B5] Andresen B. D. (1977). Synthesis of sodium formate-^13^C and oxalic acid-^13^C_2_ . J. Org. Chem. 42, 2790. 10.1021/jo00436a035

[B6] Angamuthu R. Byers P. Lutz M. Spek A. L. Bouwman E. (2010). Electrocatalytic CO _2_ conversion to oxalate by a copper complex. Sci. (1979). 327, 313–315. 10.1126/science.1177981 20075248

[B7] Banerjee A. Kanan M. W. (2018). Carbonate-promoted hydrogenation of carbon dioxide to multicarbon carboxylates. ACS Cent. Sci. 4, 606–613. 10.1021/acscentsci.8b00108 29806007 PMC5968515

[B8] Becker J. Y. Vainas B. Eger (née Levin) R. Kaufman (Née Orenstein) L. (1985). Electrocatalytic reduction of CO to oxalate by Ag and Pd porphyrins. J. Chem. Soc. Chem. Commun., 1471–1472. 10.1039/C39850001471

[B9] Clark T. Chandrasekhar J. Spitznagel G. W. Schleyer P. v. R. (1983). Efficient diffuse function-augmented basis sets for anion calculations. III. The 3-21^+^G basis set for first-row elements. Li–F. J. Comput. Chem. 4, 294–301. 10.1002/jcc.540040303

[B10] Ditchfield R. Hehre W. J. Pople J. A. (1971). Self‐Consistent molecular‐orbital methods. IX. An extended Gaussian‐type basis for molecular‐orbital studies of organic molecules. J. Chem. Phys. 54, 724–728. 10.1063/1.1674902

[B11] Dudeney A. W. L. Tarasova I. I. (1998). Photochemical decomposition of trisoxalatoiron(III):A hydrometallurgical application of daylight. Hydrometallurgy 47, 243–257. 10.1016/S0304-386X(97)00049-2

[B12] Evans W. J. Seibel C. A. Ziller J. W. (1998). Organosamarium-mediated transformations of CO_2_ and COS: monoinsertion and disproportionation reactions and the reductive coupling of CO_2_ to [O_2_CCO_2_]^2-^ . Inorg. Chem. 37, 770–776. 10.1021/ic971381t

[B13] Fenton D. M. Steinwand P. J. (1974). Noble metal catalysis. III. Preparation of dialkyl oxalates by oxidative carbonylation. J. Org. Chem. 39, 701–704. 10.1021/jo00919a026

[B14] Frisch M. J. Trucks G. W. Schlegel H. B. Scuseria G. E. Robb M. A. Cheeseman J. R. (2016). Gaussian 16, revision C.01. Wallingford CT: Gaussian, Inc.

[B15] Fukui K. (1970). Formulation of the reaction coordinate. J. Phys. Chem. 74, 4161–4163. 10.1021/j100717a029

[B16] Fukui K. (1981). The path of chemical reactions - the IRC approach. Acc. Chem. Res. 14, 363–368. 10.1021/ar00072a001

[B17] Gibson M. I. Chen P. Y. Johnson A. C. Pierce E. Can M. Ragsdale S. W. (2016). One-carbon chemistry of oxalate oxidoreductase captured by X-ray crystallography. Proc. Nat. Acad. Sci. 113, 320–325. 10.1073/pnas.1518537113 26712008 PMC4720323

[B18] Gonzalez C. Schlegel H. B. (1990). Reaction path following in mass-weighted internal coordinates. J. Phys. Chem. 94, 5523–5527. 10.1021/j100377a021

[B19] Gordon M. S. (1980). The isomers of silacyclopropane. Chem. Phys. Lett. 76, 163–168. 10.1016/0009-2614(80)80628-2

[B20] Górski A. Kraśnicka A. D. (1987). Formation of oxalates and carbonates in the thermal decompositions of alkali metal formates. J. Therm. Anal. 32, 1243–1251. 10.1007/BF01913982

[B21] Gorski A. Krasnicka A. D. (1987). Influence of the cation on the formation of free hydrogen and formaldehyde in the thermal decomposition of formates. J. Therm. Anal. 32, 1345–1354. 10.1007/BF01913334

[B22] Grimme S. (2006). Semiempirical GGA-type density functional constructed with a long-range dispersion correction. J. Comput. Chem. 27, 1787–1799. 10.1002/jcc.20495 16955487

[B23] Hariharan Pc. Pople J. A. (1973). The influence of polarization functions on molecular orbital hydrogenation energies. Theor. Chem. Acc. 28, 213–222. 10.1007/BF00533485

[B24] Hariharan Pc. Pople J. A. (1974). Accuracy of AH_n_ equilibrium geometries by single determinant molecular orbital theory. Mol. Phys. 27, 209–214. 10.1080/00268977400100171

[B25] Hehre W. J. Ditchfield R. Pople J. A. (1972). Self—consistent molecular orbital methods. XII. Further extensions of Gaussian—type basis sets for use in molecular orbital studies of organic molecules. J. Chem. Phys. 56, 2257–2261. 10.1063/1.1677527

[B26] IEA (2020). Industry direct CO2 emissions in the sustainable development scenario. Available online at: https://www.iea.org/data-and-statistics/charts/industry-direct-co2-emissions-in-the-sustainable-development-scenario-2000-2030 (Accessed February 28, 2025).

[B27] Kennedy J. J. (1938). The alkali metal cesium and some of its salts. Chem. Rev. 23, 157–163. 10.1021/cr60074a008

[B28] Krishnan R. Binkley J. Seeger R. Pople J. A. (1980). Self‐consistent molecular orbital methods. XX. A basis set for correlated wave functions. J. Chem. Phys. 72, 650–654. 10.1063/1.438955

[B29] Kushi Y. Nagao H. Nishioka T. Isobe K. Tanaka K. (1994). Oxalate Formation in electrochemical CO_2_ reduction catalyzed by rhodium-sulfur cluster. Chem. Lett. 23, 2175–2178. 10.1246/cl.1994.2175

[B30] Kushi Y. Nagao H. Nishioka T. Isobe K. Tanaka K. (1995). Remarkable decrease in overpotential of oxalate formation in electrochemical CO_2_ reduction by a metal–sulfide cluster J. Chem. Soc. Chem. Commun. J. Chem. Soc. Chem. Commun. 12, 1223–1224. 10.1039/C39950001223

[B31] Lakkaraju P. S. Askerka M. Beyer H. Ryan C. T. Dobbins T. Bennett C. (2016). Formate to oxalate: a crucial step for the conversion of carbon dioxide into multi-carbon compounds. ChemCatChem 8, 3453–3457. 10.1002/cctc.201600765

[B32] Lee S. Oh J. Shin B., (1999). Dissolution of iron oxide rust materials using oxalic acid, 115, 815–819. 10.2473/shigentosozai.115.815

[B33] Lew K. (2009). Acids and bases. Infobase Publishing. Available online at: https://www.amazon.com/Acids-Bases-Essential-Chemistry-Kristi/dp/0791097838.

[B34] McLean A. D. Chandler G. S. (1980). Contracted Gaussian basis sets for molecular calculations. I. Second row atoms, Z=11–18. J. Chem. Phys. 72, 5639–5648. 10.1063/1.438980

[B35] Miertuš S. Scrocco E. Tomasi J. (1981). Electrostatic interaction of a solute with a continuum. A direct utilizaion of *ab initio* molecular potentials for the prevision of solvent effects. Chem. Phys. 55, 117–129. 10.1016/0301-0104(81)85090-2

[B36] Morando P. J. Piacquadio N H. Blesa M. A. Vedova C. O. D. (1987). The thermal decomposition of iron(III) formate. Thermochim. Acta 117, 325–330. 10.1016/0040-6031(87)88126-1

[B37] Ogi Y. Obara Y. Katayama T. Suzuki Y.-I. Liu S. Y. Bartlett N. C.-M. (2015). Ultraviolet photochemical reaction of [Fe(III)(C_2_O_4_)_3_]^3-^ in aqueous solutions studied by femtosecond time-resolved X-ray absorption spectroscopy using an X-ray free electron laser. Struct. Dyn. 2, 034901. 10.1063/1.4918803 26798796 PMC4711623

[B38] Parker C. A. Hatchard C. G. (1959). Photodecomposition of complex oxalates–some preliminary experiments by flash photolysis. J. Phys. Chem. 63, 22–26. 10.1021/j150571a009

[B39] Pastero A. Curetti N. Ortenzi M. A. M S. Destefanis E. Pavese A. (2019). CO_2_ capture and sequestration in stable Ca-oxalate, via Ca-ascorbate promoted green reaction. Sci. Total Environ. 666, 1232–1244. 10.1016/j.scitotenv.2019.02.114 30970488

[B40] Prieto G. (2017). Carbon dioxide hydrogenation into higher hydrocarbons and oxygenates: thermodynamic and kinetic bounds and progress with heterogeneous and homogeneous catalysis. ChemSusChem 10, 1056–1070. 10.1002/cssc.201601591 28247481

[B41] Riemenschneider W. Tanifuji M. (2011). “Oxalic acid,” in Ullmann's encyclopedia of industrial chemistry (Weinheim: Wiley-VCH Verl.).

[B42] Santawaja P. Kudo S. Mori A. Tahara A. Asano S. Hayashi J. (2020). Sustainable iron-making using oxalic acid: the concept, A brief review of key reactions, and an experimental demonstration of the iron-making process. ACS Sus. Chem. Eng. 8, 13292–13301. 10.1021/acssuschemeng.0c03593

[B43] Santawaja P. Kudo S. Tahara A. Asano S. Hayashi J. (2022). Dissolution of iron oxides highly loaded in oxalic acid aqueous solution for a potential application in iron-making. ISIJ Int. 62, 2466–2475. 10.2355/isijinternational.isijint-2020-726

[B44] Savéant J. M. (2008). Molecular catalysis of electrochemical reactions. Mechanistic aspects. Chem. Rev. 108, 2348–2378. 10.1021/cr068079z 18620367

[B45] Schuler E. Demetriou M. Shiju N. R. Gruter G. J. M. (2021). Towards sustainable oxalic acid from CO_2_ and biomass. ChemSusChem 14, 3636–3664, and references therein. 10.1002/cssc.202101272 34324259 PMC8519076

[B46] Schuler E. Ermolich P. A. Shiju N. R. Gruter G. J. M. (2021a). Monomers from CO_2_: superbases as catalysts for formate-to-oxalate coupling. ChemSusChem 14, 1517–1523. 10.1002/cssc.202002725 33427392 PMC8048464

[B47] Schuler E. Stoop M. Shiju N. R. Gruter G. J. M. (2021b). Stepping stones in CO2 utilization: optimizing the formate to oxalate coupling reaction using response surface modeling. ACS Sus. Chem. Eng. 9, 14777–14788. 10.1021/acssuschemeng.1c04539 PMC8579406 34777925

[B48] Shishido S. Masuda Y. (1971). Thermogravimetric analysis of various formates. Nikkashi 92, 309–312. 10.1246/nikkashi1948.92.309

[B49] Stephens P. J. Devlin F. J. Chabalowski C. F. Frisch M. J. (1994). *Ab initio* calculation of vibrational absorption and circular dichroism spectra using density functional force fields. J. Phys. Chem. 98, 11623–11627. 10.1021/j100096a001

[B50] Viertelhaus M. Adler P. Cle´rac R. Anson C. E. Powell A. K. (2005). Iron(II) formate [Fe(O_2_CH)_2_]·1/3HCO_2_H: a mesoporous magnet–solvothermal syntheses and crystal structures of the isomorphous framework metal(II) formates [M(O_2_CH)_2_]·*n*(Solvent) (M = Fe, Co, Ni, Zn, Mg). Eur. J. Inorg. Chem. 2005, 692–703. 10.1002/ejic.200400395

[B51] Watanabe R. Yamauchi M. Sadakiyo M. Abe R. Takeguchi T. (2015). CO_2_-free electric power circulation via direct charge and discharge using the glycolic acid/oxalic acid redox couple. Energy Environ. Sci. 8, 1456–1462. 10.1039/c5ee00192g

[B52] Zhang E. Noble A. Ji B. Li Q. (2022). Effects of contaminant metal ions on precipitation recovery of rare earth elements using oxalic acid. J. Rare Earths. 40, 482–490. 10.1016/j.jre.2020.11.008

[B53] Zuo J. Gao L. Fan L. (2016). Amelioration of postharvest chilling injury in sweet pepper by glycine betaine. Postharv. Biol. Technol. 112, 114–120. 10.1016/j.postharvbio.2015.07.008

