# Supplementary material for: Effect of alkali metal cations on dehydrogenative coupling of formate anions to oxalate
Source: Front Chem. 2025 Apr 23;13:1588773. doi: 10.3389/fchem.2025.1588773 (PMC12055785; doi:10.3389/fchem.2025.1588773)
Supplement: Supplementary file 1 [file Presentation1.pdf]

Supplementary Material

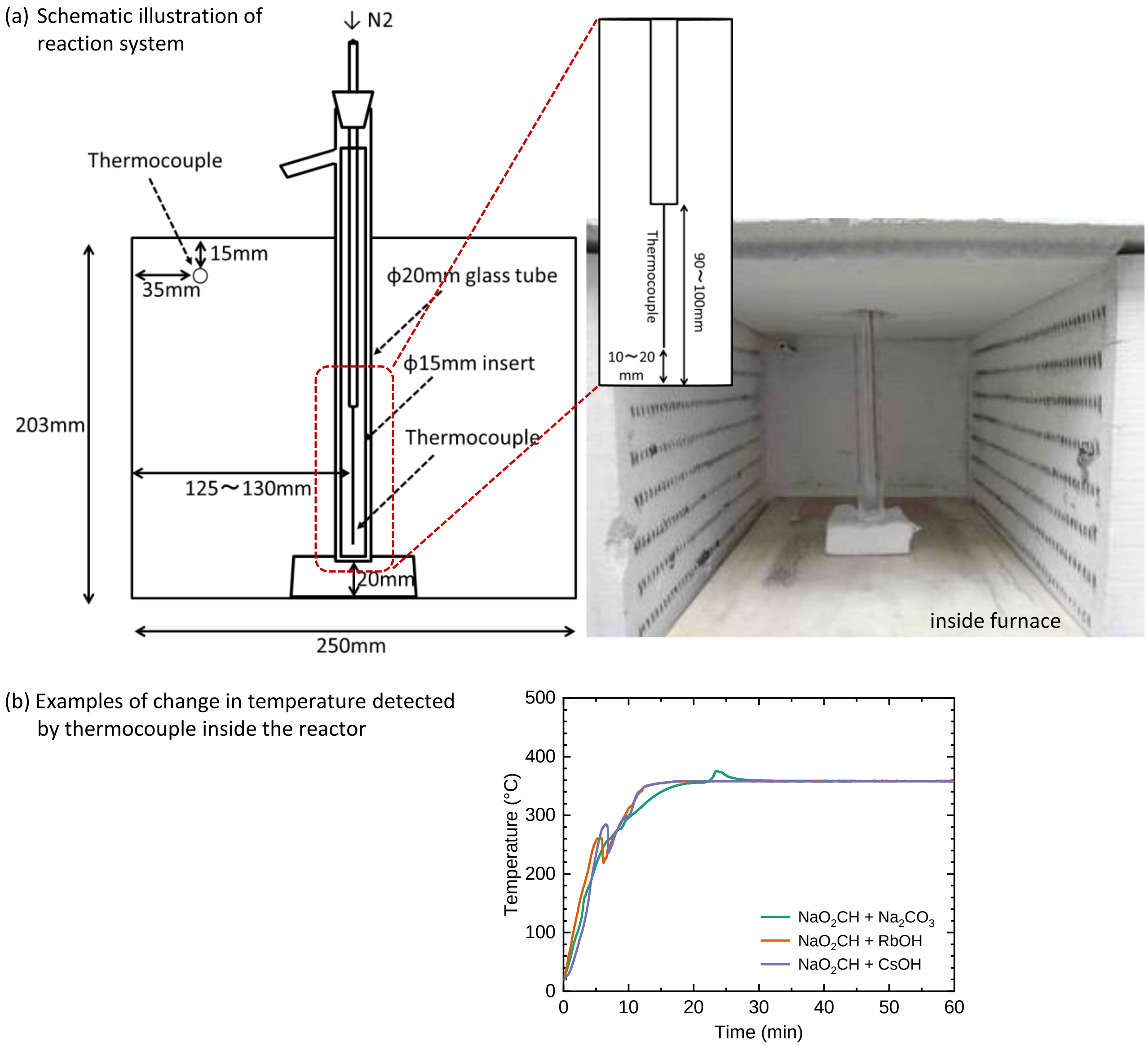

Supplementary Figure 1. Reactor used for thermal coupling of formate to oxalate. The reactor loaded with 45 mmol of formate (and base) at the bottom was inserted to a muffle furnace heated to a prescribed temperature, typically 360 °C. Sample temperature was monitored with a thermocouple inserted in the bed as shown in Supplementary Figure S1 (b). Upward and downward shoulder peaks in the profiles show endothermic (melting of formate) and exothermic (formate coupling) events, respectively.

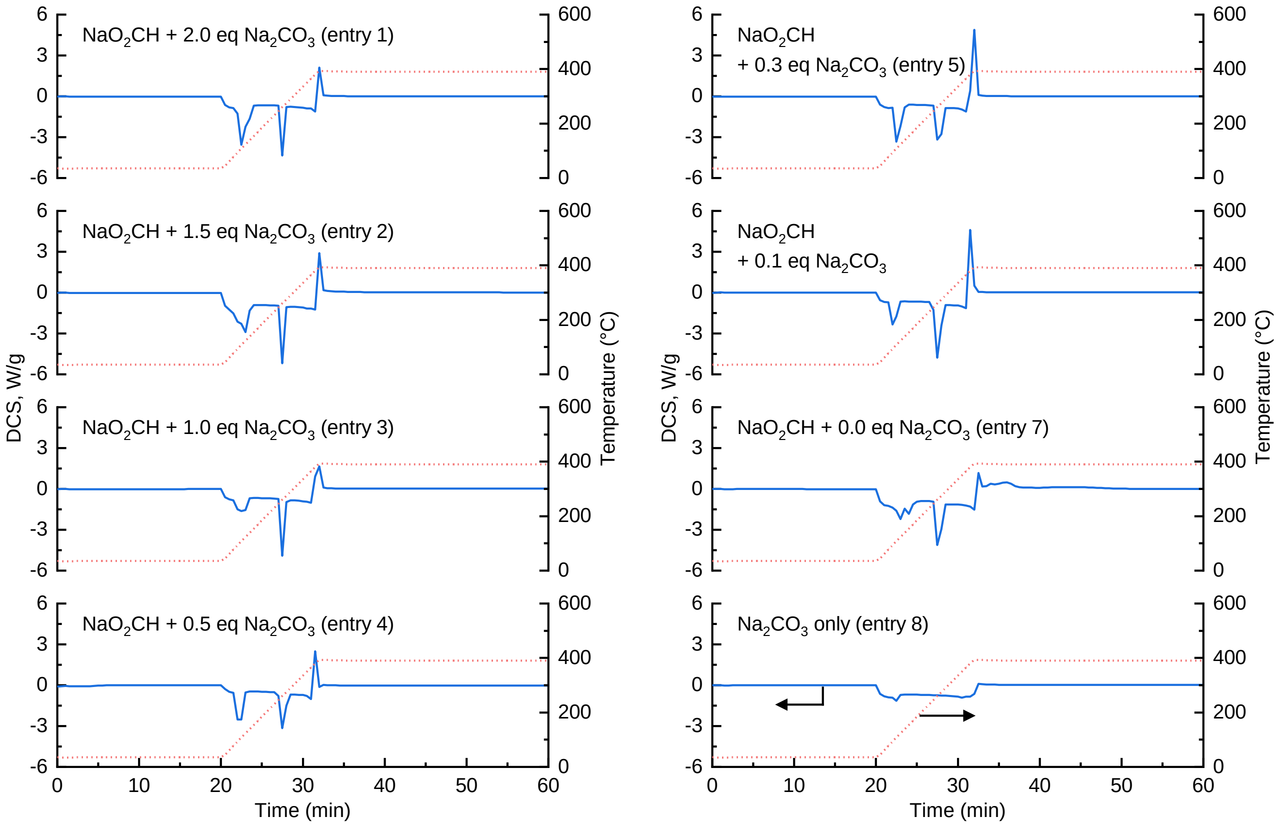

Supplementary Figure 2. DSC profile of sodium formate/sodium carbonate system (Table 1). Measurement temp; 30-390 °C, heating rate; 30 °C/min.

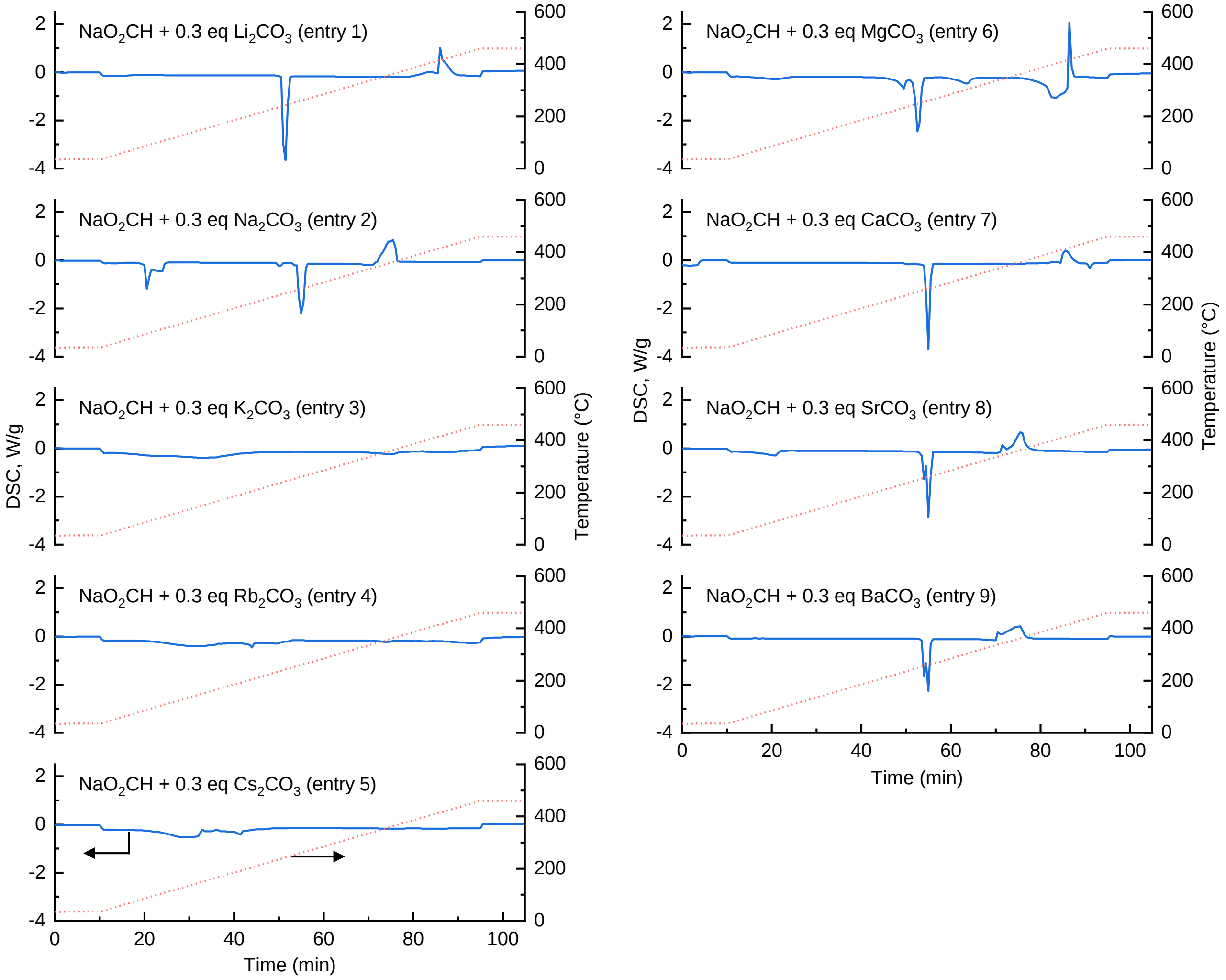

Supplementary Figure 3. DCS profile of sodium formate/0.3 eq. metal carbonate system (Table 2). Measurement temp; 30-460 °C, heating rate; 5 °C/min.

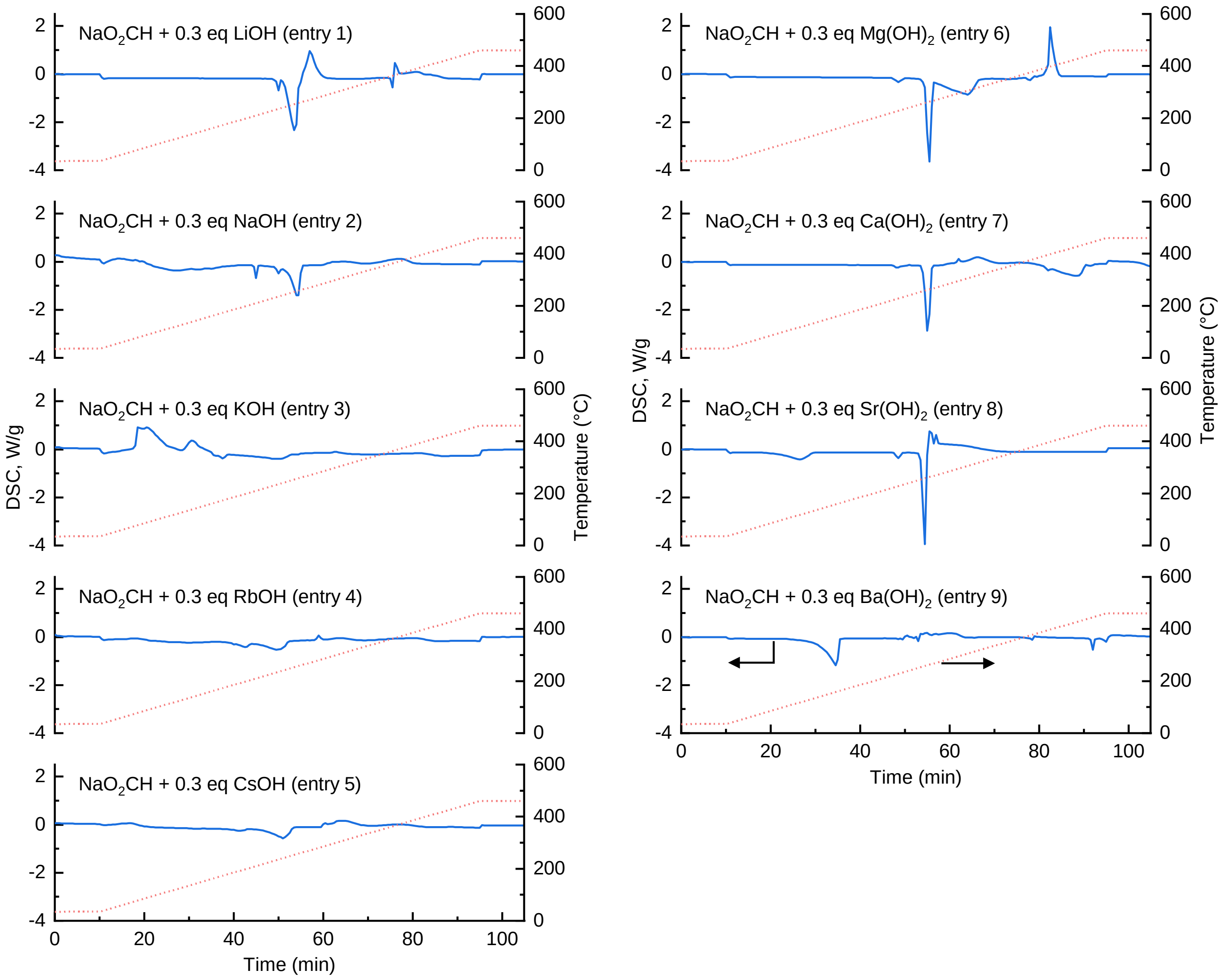

Supplementary Figure 4. DSC profile of sodium formate/0.3 eq. metal hydroxide system (Table 3). Measurement temp; 30-460 °C, heating rate; 5 °C/min.

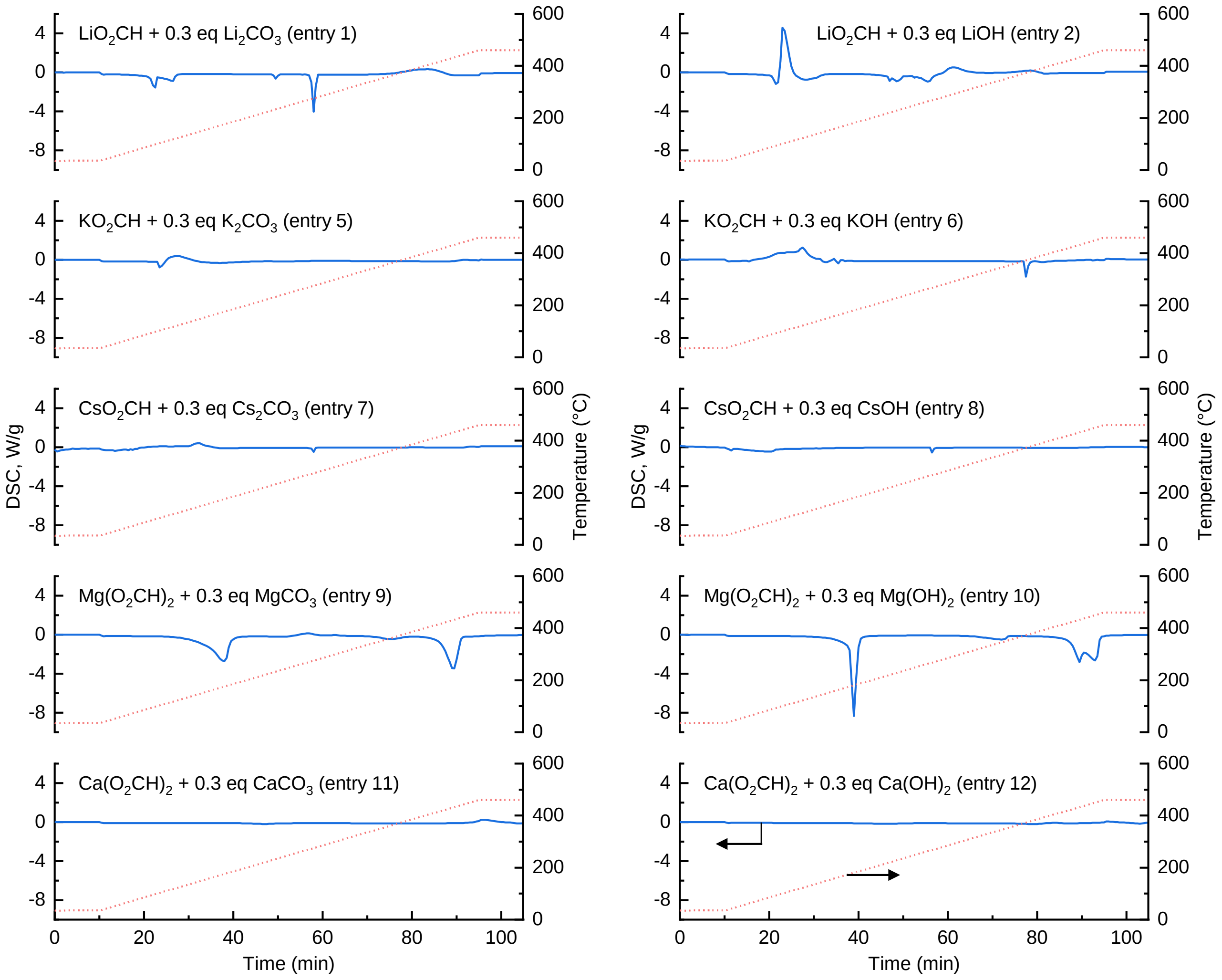

Supplementary Figure 5. DSC profile of metal formate/0.3 eq. metal carbonate or hydroxide system (Table 4). Measurement temp; 30-460 °C, heating rate; 5 °C/min.

Supplementary Table 1. Optimized structures with relative gibbs free energies [kcal/mol] (part 1/9)

M^1 = Na, M^2 = Li	M^1 = M^2 = Na	M^1 = Na, M^2 = K	M^1 =Na, M^2 =Rb	M^1 =Na, M^2 =Cs
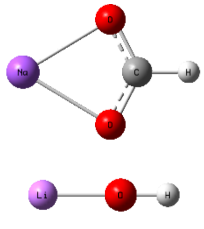	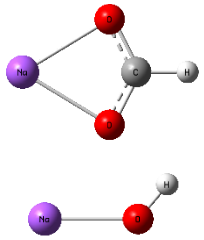	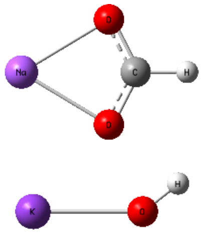	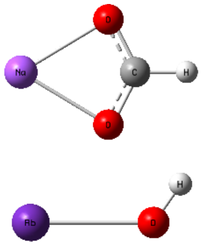	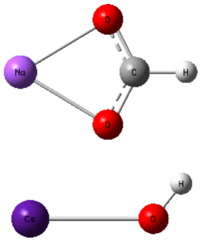
**Formate-Na + LiOH (0.0)**	**Formate-Na + NaOH (0.0)**	**Formate-Na + KOH (0.0)**	**Formate-Na + RbOH (0.0)**	**Formate-Na + CsOH (0.0)**
Converged to **$A1c^{NaLi}$**	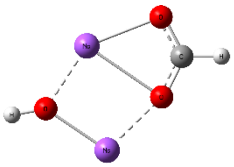	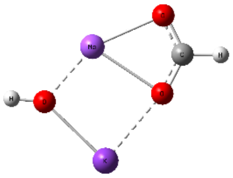	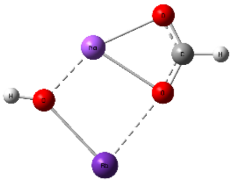	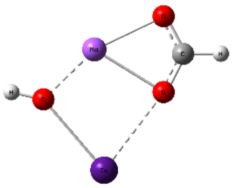
**$A1a^{NaLi}$ (–)**	**$A1a^{NaNa}$ (–4.1)**	**$A1a^{NaK}$ (–3.2)**	**$A1a^{NaRb}$ (–4.9)**	**$A1a^{NaCs}$ (–5.2)**
Converged to **$A1c^{NaLi}$**	Same as **$A1a^{NaNa}$**	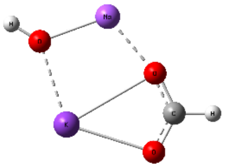	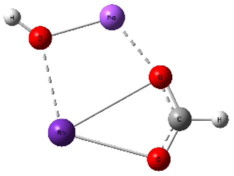	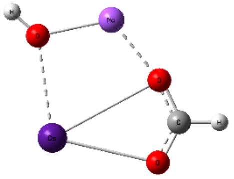
**$A1b^{NaLi}$ (–)**	**$A1b^{NaNa}$ (–4.1)**	**$A1b^{NaK}$ (–3.0)**	**$A1b^{NaRb}$ (–4.3)**	**$A1b^{NaCs}$ (–3.8)**
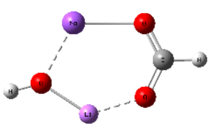	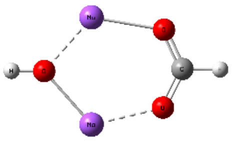	Converged to **$A1b^{NaK}$**	Converged to **$A1b^{NaRb}$**	Converged to **$A1b^{NaCs}$**
**$A1c^{NaLi}$ (–12.1)**	**$A1c^{NaNa}$ (–5.9)**	**$A1c^{NaK}$ (–)**	**$A1c^{NaRb}$ (–)**	**$A1c^{NaCs}$ (–)**

Supplementary Table 2. Optimized structures with relative gibbs free energies [kcal/mol] (part 2/9)

M^1 = Na, M^2 = Li	M^1 = M^2 = Na	M^1 = Na, M^2 = K	M^1 =Na, M^2 =Rb	M^1 =Na, M^2 =Cs
could not<br>be converged	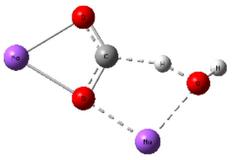	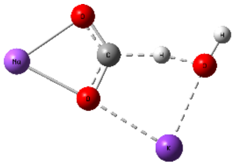	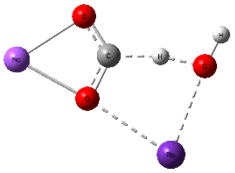	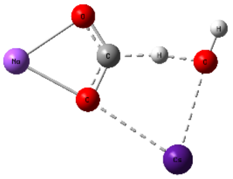
**$TS_{A1/A2}^{NaLi}$**<br>**(–)**	**$TS_{A1/A2}^{NaNa}$**<br>**(+30.7)**	**$TS_{A1/A2}^{NaK}$**<br>**(+27.4)**	**$TS_{A1/A2}^{NaRb}$**<br>**(+24.9)**	**$TS_{A1/A2}^{NaCs}$**<br>**(+24.6)**
could not<br>be converged	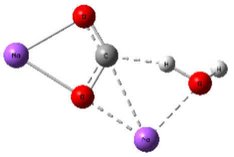	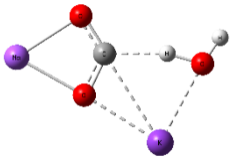	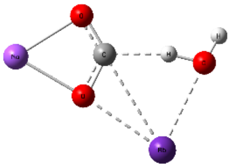	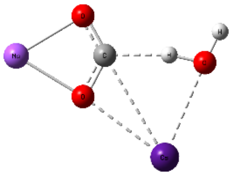
**$A2^{NaLi}$**<br>**(–)**	**$A2^{NaNa}$**<br>**(+32.2)**	**$A2^{NaK}$**<br>**(+32.3)**	**$A2^{NaRb}$**<br>**(+30.8)**	**$A2^{NaCs}$**<br>**(+30.1)**
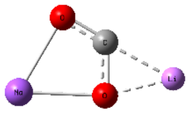	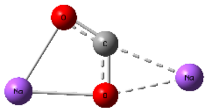	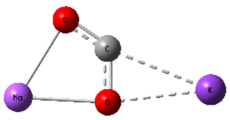	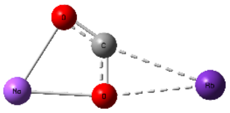	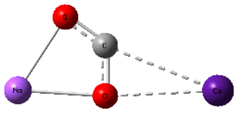
**Carbonite**<br>**-NaLi + H_2O**<br>**(+29.9)**	**Carbonite**<br>**-NaNa + H_2O**<br>**(+33.3)**	**Carbonite**<br>**-NaK + H_2O**<br>**(+34.0)**	**Carbonite**<br>**-NaRb + H_2O**<br>**(+33.8)**	**Carbonite**<br>**-NaCs + H_2O**<br>**(+34.5)**
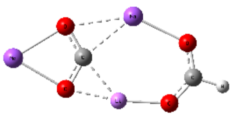	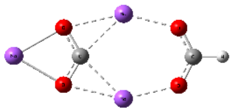	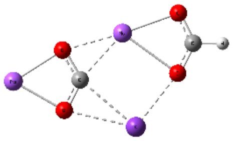	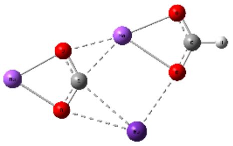	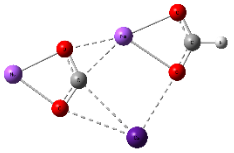
**$A3^{NaLi}$**<br>**(+24.6)**	**$A3^{NaNa}$**<br>**(+30.2)**	**$A3^{NaK}$**<br>**(+31.6)**	**$A3^{NaRb}$**<br>**(+29.9)**	**$A3^{NaCs}$**<br>**(+30.4)**

Supplementary Table 3. Optimized structures with relative gibbs free energies [kcal/mol] (part 3/9)

M^1 = Na, M^2 = Li	M^1 = M^2 = Na	M^1 = Na, M^2 = K	M^1 =Na, M^2 =Rb	M^1 =Na, M^2 =Cs
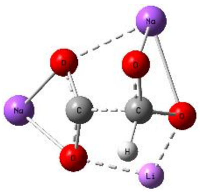	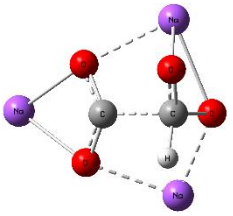	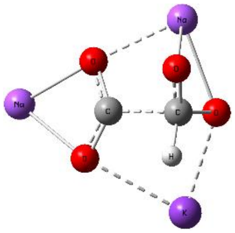	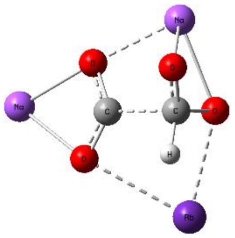	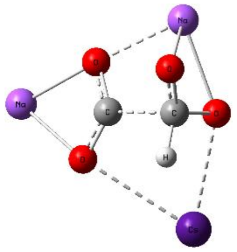
$TS_{A3/A4}^{NaLi}$ (+50.7)	$TS_{A3/A4}^{NaNa}$ (+55.9)	$TS_{A3/A4}^{NaK}$ (+55.5)	$TS_{A3/A4}^{NaRb}$ (+53.7)	$TS_{A3/A4}^{NaCs}$ (+53.8)
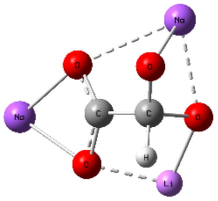	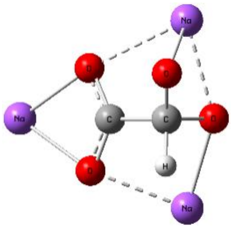	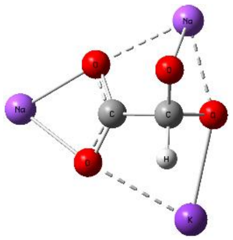	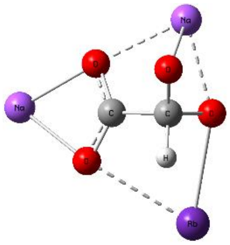	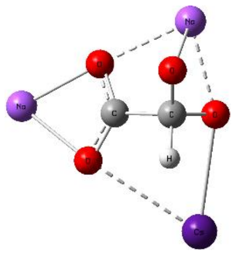
$A4^{NaLi}$ (+40.8)	$A4^{NaNa}$ (+46.5)	$A4^{NaK}$ (+46.9)	$A4^{NaRb}$ (+45.3)	$A4^{NaCs}$ (+45.5)
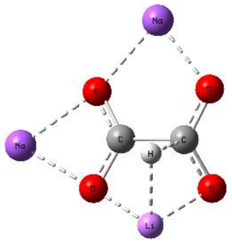	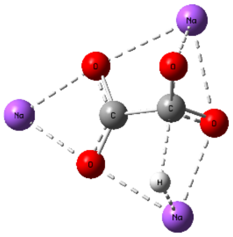	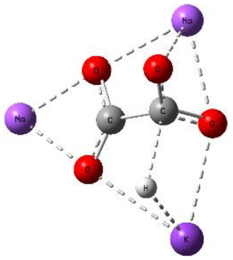	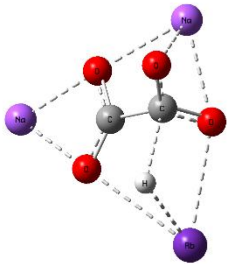	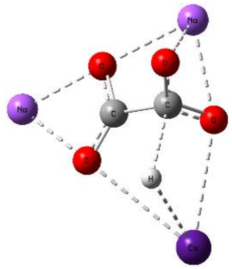
$TS_{A4/A5}^{NaLi}$ (+49.1)	$TS_{A4/A5}^{NaNa}$ (+60.8)	$TS_{A4/A5}^{NaK}$ (+61.7)	$TS_{A4/A5}^{NaRb}$ (+60.7)	$TS_{A4/A5}^{NaCs}$ (+61.3)

Supplementary Table 4. Optimized structures with relative gibbs free energies [kcal/mol] (part 4/9)

M^1 = Na, M^2 = Li	M^1 = M^2 = Na	M^1 = Na, M^2 = K	M^1 =Na, M^2 =Rb	M^1 =Na, M^2 =Cs
Converged to **A6^{NaLi}**	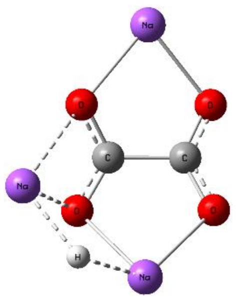	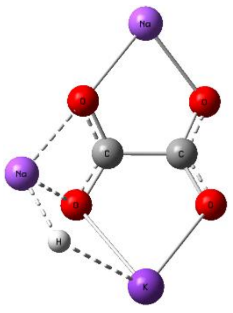	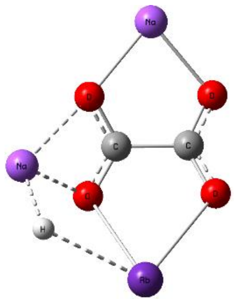	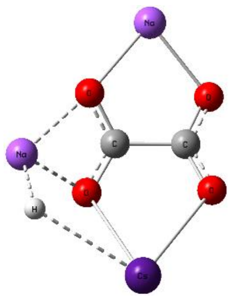
**A5^{NaLi}**<br>**(–)**	**A5^{NaNa}**<br>**(+27.9)**	**A5^{NaK}**<br>**(+27.7)**	**A5^{NaRb}**<br>**(+25.9)**	**A5^{NaCs}**<br>**(+25.9)**
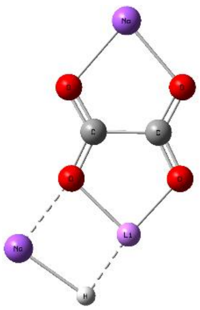	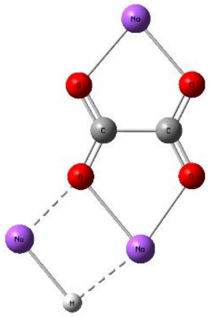	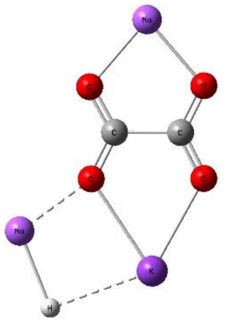	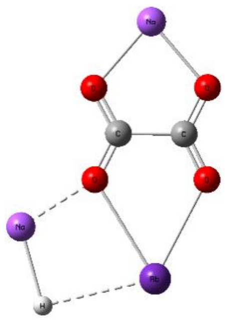	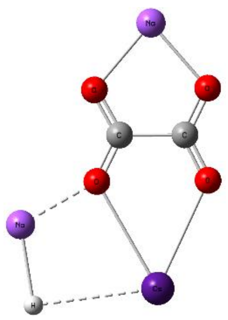
**A6^{NaLi}**<br>**(+16.9)**	**A6^{NaNa}**<br>**(+22.6)**	**A6^{NaK}**<br>**(+23.7)**	**A6^{NaRb}**<br>**(+22.2)**	**A6^{NaCs}**<br>**(+22.4)**
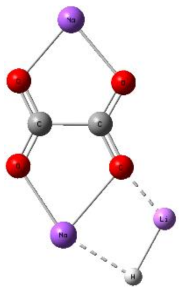	Same as **A6^{NaNa}**	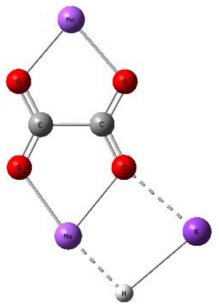	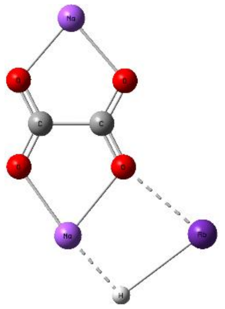	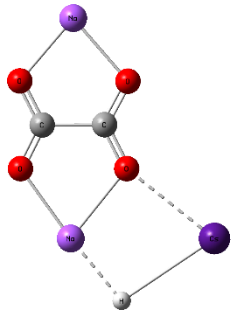
**A7^{NaLi}**<br>**(+19.9)**	**A7^{NaNa}**<br>**(–)**	**A7^{NaK}**<br>**(+22.5)**	**A7^{NaRb}**<br>**(+20.6)**	**A7^{NaCs}**<br>**(+20.9)**

Supplementary Table 5. Optimized structures with relative gibbs free energies [kcal/mol] (part 5/9)

M^1 = Na, M^2 = Li	M^1 = M^2 = Na	M^1 = Na, M^2 = K	M^1 =Na, M^2 =Rb	M^1 =Na, M^2 =Cs
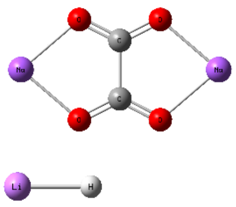	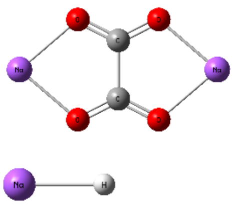	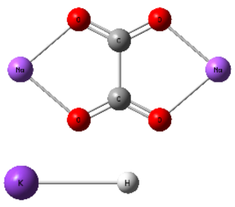	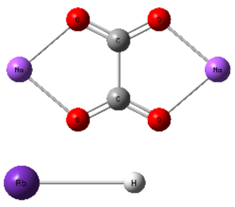	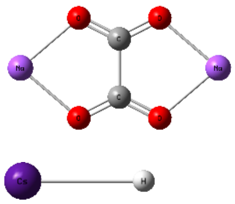
**Oxalate-NaNa + LiH (+20.7)**	**Oxalate-NaNa + NaH (+21.6)**	**Oxalate-NaNa + KH (+21.1)**	**Oxalate-NaNa + RbH (+19.4)**	**Oxalate-NaNa + CsH (+19.7)**
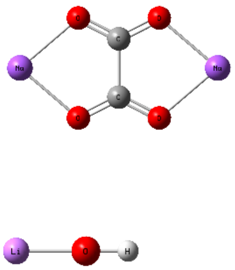	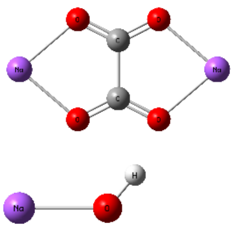	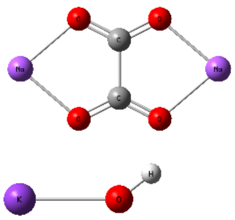	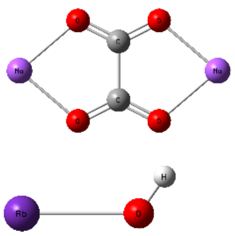	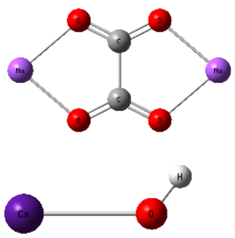
**Oxalate-NaNa + LiOH + H_2 (+5.5)**	**Oxalate-NaNa + NaOH + H_2 (+5.5)**	**Oxalate-NaNa + KOH + H_2 (+5.5)**	**Oxalate-NaNa + RbOH + H_2 (+5.5)**	**Oxalate-NaNa + CsOH + H_2 (+5.5)**

Supplementary Table 6. Optimized structures with relative gibbs free energies [kcal/mol] (part 6/9)

M^1 = Li, M^2 =Na	$M^1 = M^2$ = Li	$M^1 = M^2$ = K	$M^1 = M^2$ = Rb	$M^1 = M^2$ = Cs
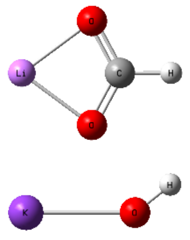	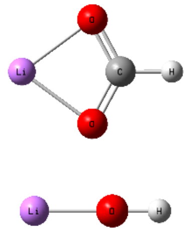	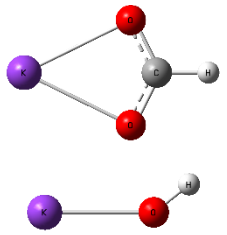	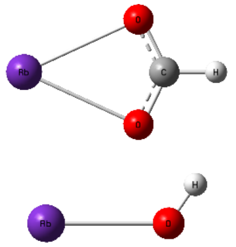	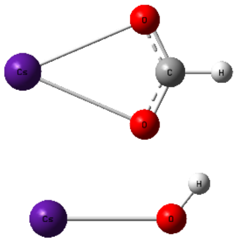
**Formate-Li + NaOH (0.0)**	**Formate-Li + LiOH (0.0)**	**Formate-K + KOH (0.0)**	**Formate-Rb + RbOH (0.0)**	**Formate-Cs + CsOH (0.0)**
Same as **$A1c^{NaLi}$**	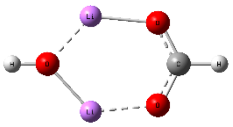	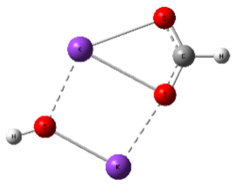	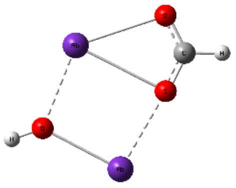	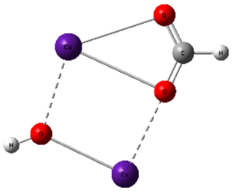
**$A1^{LiNa}$ (–12.1)**	**$A1^{LiLi}$ (–18.4)**	**$A1^{KK}$ (+0.2)**	**$A1^{RbRb}$ (–2.0)**	**$A1^{CsCs}$ (–0.1)**
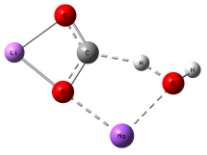	could not be converged	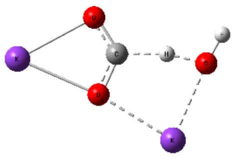	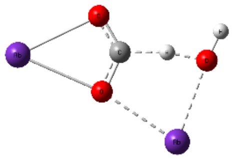	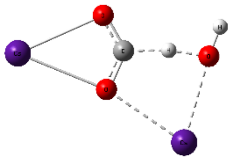
**$TS_{A1/A2}^{LiNa}$ (+28.4)**	**$TS_{A1/A2}^{LiLi}$ (–)**	**$TS_{A1/A2}^{KK}$ (+30.8)**	**$TS_{A1/A2}^{RbRb}$ (+27.8)**	**$TS_{A1/A2}^{CsCs}$ (+28.3)**
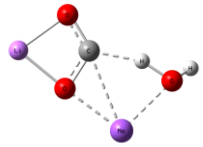	could not be converged	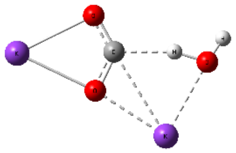	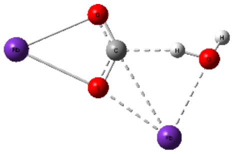	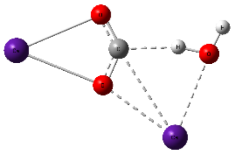
**$A2^{LiNa}$ (+28.5)**	**$A2^{LiLi}$ (–)**	**$A2^{KK}$ (+36.5)**	**$A2^{RbRb}$ (+35.7)**	**$A2^{CsCs}$ (+38.0)**

Supplementary Table 7. Optimized structures with relative gibbs free energies [kcal/mol] (part 7/9)

M^1 = Li, M^2 =Na	M^1 = M^2 = Li	M^1 = M^2 = K	M^1 = M^2 = Rb	M^1 = M^2 = Cs
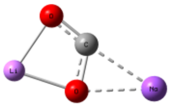	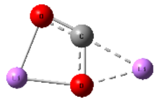	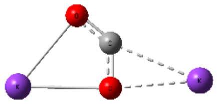	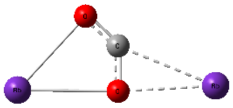	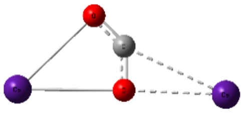
Carbonite -LiNa + H_2O (+30.5)	Carbonite -LiLi + H_2O (+26.6)	Carbonite -KK + H_2O (+38.4)	Carbonite -RbRb + H_2O (+39.8)	Carbonite -CsCs + H_2O (+43.1)
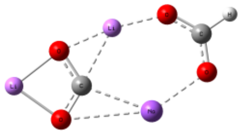	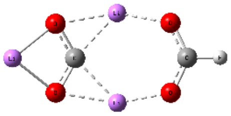	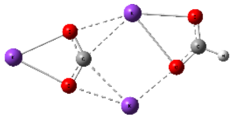	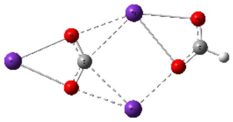	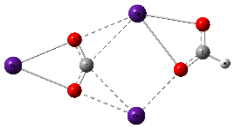
$A3^{LiNa}$ (+20.1)	$A3^{LiLi}$ (+18.7)	$A3^{KK}$ (+39.4)	$A3^{RbRb}$ (+37.8)	$A3^{CsCs}$ (+41.7)
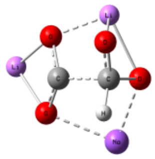	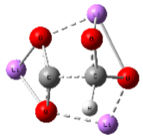	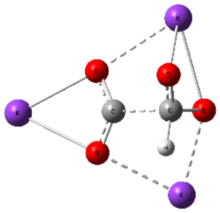	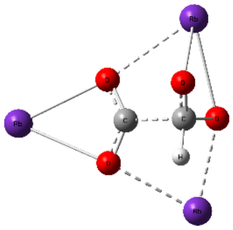	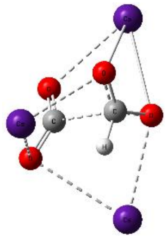
$TS_{A3/A4}^{LiNa}$ (+50.1)	$TS_{A3/A4}^{LiLi}$ (+45.8)	$TS_{A3/A4}^{KK}$ (+60.8)	$TS_{A3/A4}^{RbRb}$ (+57.8)	$TS_{A3/A4}^{CsCs}$ (+68.2)
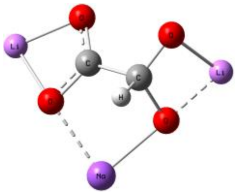	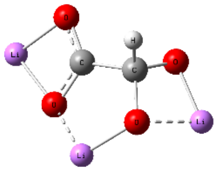	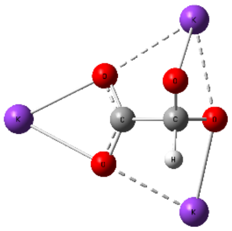	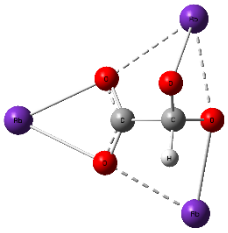	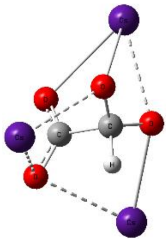
$A4^{LiNa}$ (+33.5)	$A4^{LiLi}$ (+23.6)	$A4^{KK}$ (+54.4)	$A4^{RbRb}$ (+53.7)	$A4^{CsCs}$ (+54.7)

Supplementary Table 8. Optimized structures with relative gibbs free energies [kcal/mol] (part 8/9)

M^1 = Li, M^2 =Na	M^1 = M^2 = Li	M^1 = M^2 = K	M^1 = M^2 = Rb	M^1 = M^2 = Cs
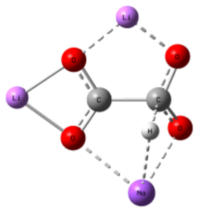	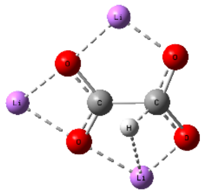	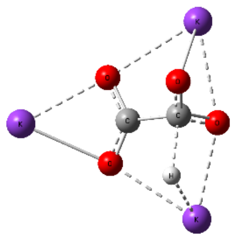	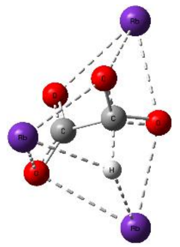	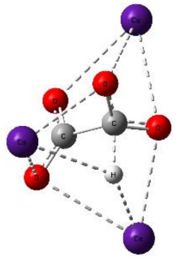
$TS_{A4/A5}^{LiNa}$ (+45.2)	$TS_{A4/A5}^{LiLi}$ (+40.2)	$TS_{A4/A5}^{KK}$ (+65.3)	$TS_{A4/A5}^{RbRb}$ (+63.1)	$TS_{A4/A5}^{CsCs}$ (+64.5)
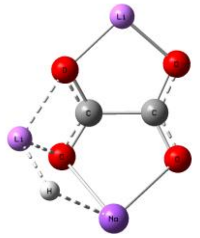	Converged to $A6^{LiLi}$	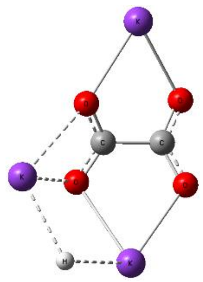	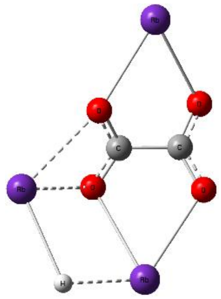	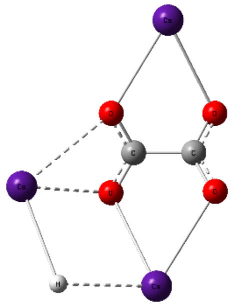
$A5^{LiNa}$ (+23.6)	$A5^{LiLi}$ (–)	$A5^{KK}$ (+30.5)	$A5^{RbRb}$ (+27.8)	$A5^{CsCs}$ (+29.0)
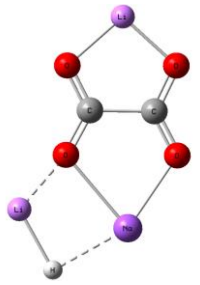	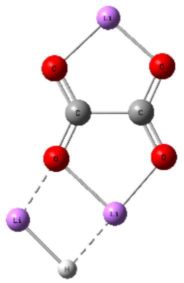	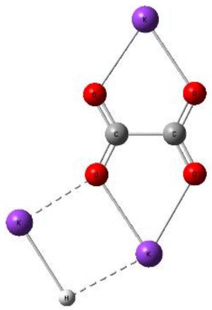	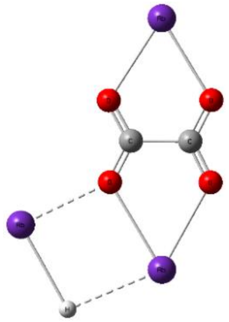	Converged to $A5^{CsCs}$
$A6^{LiNa}$ (+15.4)	$A6^{LiLi}$ (+10.9)	$A6^{KK}$ (+29.4)	$A6^{RbRb}$ (+27.3)	$A6^{CsCs}$ (–)

Supplementary Table 9. Optimized structures with relative gibbs free energies [kcal/mol] (part 9/9)

M^1 = Li, M^2 =Na	M^1 = M^2 = Li	M^1 = M^2 = K	M^1 = M^2 = Rb	M^1 = M^2 = Cs
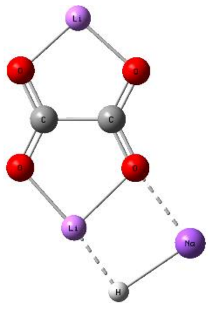	Same as<br>$A6^{LiLi}$	Same as<br>$A6^{LiLi}$	Same as<br>$A6^{LiLi}$	Same as<br>$A6^{LiLi}$
$A7^{LiNa}$<br>(+12.8)	$A7^{LiLi}$<br>(–)	$A7^{KK}$<br>(–)	$A7^{RbRb}$<br>(–)	$A7^{CsCs}$<br>(–)
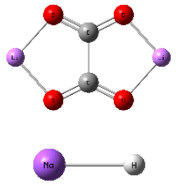	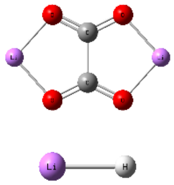	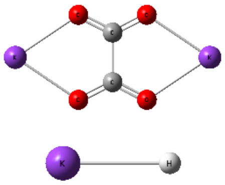	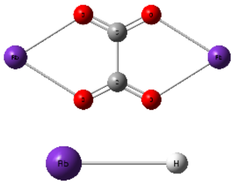	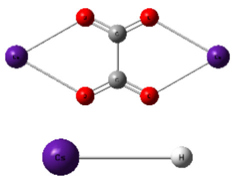
Oxalate-LiLi<br>+ NaH (+14.1)	Oxalate-LiLi<br>+ LiH (+13.1)	Oxalate-KK<br>+ KH (+25.1)	Oxalate-RbRb<br>+ RbH (+24.3)	Oxalate-CsCs<br>+ CsH (+25.1)
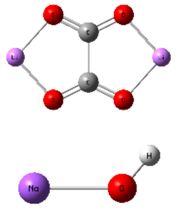	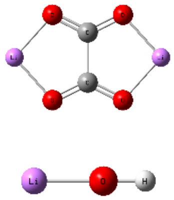	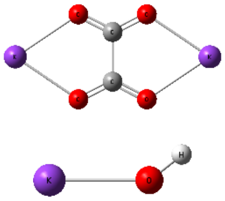	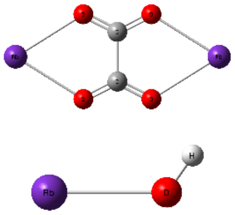	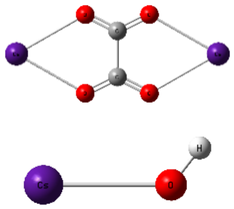
Oxalate-LiLi<br>+ NaOH + H_2<br>(+0.0)	Oxalate-LiLi<br>+ LiOH + H_2<br>(–2.1)	Oxalate-KK<br>+ KOH + H_2<br>(+9.6)	Oxalate-RbRb<br>+ RbOH + H_2<br>(+10.3)	Oxalate-CsCs<br>+ CsOH + H_2<br>(+10.9)

Supplementary Table 10. Thermal Parameters (Part 1/2)

Molecules / Species	Estimated parameters after optimization / frequency under BS1[a]				SCF [Hartree] for single point calculation under BS2[b]
	SCF [Hartree]	Thermal Correction [Hartree]		Imaginary Freq-uency [cm^{-1}] (*only for TS)	
		Zero point energy	Gibbs free Energy		
H_2	-76.41973740	0.021370	0.003713	–	-76.46613833
H_2O	-1.178539357	0.010174	-0.001314	–	-1.179716201
Li formate	-196.7545537	0.024028	-0.001385	–	-196.8649251
Na formate	-351.5104124	0.022981	-0.003907	–	-351.6282882
K formate	-217.5369387	0.022402	-0.005640	–	-217.6586285
Rb formate	-213.3149298	0.022187	-0.007028	–	-213.4353604
Cs formate	-209.3626043	0.021982	-0.008139	–	-209.4855229
LiOH	-83.37132223	0.012157	-0.001558	–	-83.46041986
NaOH	-238.1092580	0.010805	-0.011973	–	-238.2158557
KOH	-104.1265146	0.010142	-0.013723	–	-104.2434891
RbOH	-99.90263622	0.010142	-0.014940	–	-100.0189191
CsOH	-95.94790004	0.009971	-0.015876	–	-96.06938349
LiH	-8.082574865	0.003164	-0.012937	–	-8.143368151
NaH	-162.8519094	0.002668	-0.015355	–	-162.9052926
KH	-28.87621523	0.002182	-0.016976	–	-28.93398431
RbH	-24.65533444	0.002046	-0.018328	–	-24.71191565

CsH	-20.70212469	0.001850	-0.019303	–	-20.76193094
Li_2 oxalate	-392.3581501	0.030523	-0.000026	–	-392.5549209
Na_2 oxalate	-701.8500853	0.027423	-0.006421	–	-702.0681989
K_2 oxalate	-433.8913459	0.026116	-0.010677	–	-434.1215531
Rb_2 oxalate	-425.4430004	0.025531	-0.014013	–	-425.6732668
Cs_2 oxalate	-417.5352716	0.025190	-0.016170	–	-417.7727473
[NaLi][carbonite]	-358.4293740	0.012657	0.018859	–	-358.5685694
[NaNa][carbonite]	-513.1824124	0.011316	0.018146	–	-513.3257813
[NaK][carbonite]	-379.2074584	0.010817	0.017976	–	-379.3523179
[NaRb][carbonite]	-374.9858630	0.010608	0.017943	–	-375.1276292
[NaCs][carbonite]	-371.0343977	0.010502	0.017931	–	-371.176915
[LiNa][carbonite]	-358.4262969	0.012321	0.018632	–	-358.5670065
[LiLi][carbonite]	-203.6742485	0.013658	0.019402	–	-203.8103248
[KK][carbonite]	-245.2314848	0.010411	0.017943	–	-245.3754815
[RbRb][carbonite]	-236.7895335	0.010076	0.017961	–	-236.9248947
[CsCs][carbonite]	-228.8856453	0.009877	0.018009	–	-229.0201073

[a]BS1: B3LYP / SDD (Li, Na, K, Rb, Cs), 6-31G* (C, H, O)
[b]BS2: B3LYP-D / SDD (Li, Na, K, Rb, Cs), 6-311++G** (C, H, O) / PCM (ε = 26.0 for molten salt)

Supplementary Table 11. Thermal Parameters (Part 2/2)

Molecules / Species		Estimated parameters after optimization / frequency under BS1[a]				SCF [Hartree] for single point calculation under BS2[b]
		SCF [Hartree]	Thermal Correction [Hartree]		Imaginary Frequency [cm^{-1}] (*only for TS)	
			Zero point energy	Gibbs free Energy		
M^1 || Na M^2 || Li	**A1a^{NaLi}**	*[converged to* ***A1c^{NaLi}*** *]*				
	**A1b^{NaLi}**	*[converged to* ***A1c^{NaLi}*** *]*				
	**A1c^{NaLi}**	-434.9732189	0.038372	0.005934	–	-435.1193942
	**TS$_{A1/A2}^{NaLi}$**	*[not converged]*				
	**A2^{NaLi}**	*[converged to* ***A1c^{NaLi}*** *]*				
	**A3^{NaLi}**	-710.0219988	0.037159	-0.001711	–	-710.2230871
	**TS$_{A3/A4}^{NaLi}$**	-709.9556682	0.037526	0.002985	-290.4787	-710.1860800
	**A4^{NaLi}**	-709.9624647	0.039160	0.004222	–	-710.2032146
	**TS$_{A4/A5}^{NaLi}$**	-709.9331715	0.033760	-0.001306	-739.6566	-710.1843725
	**A5^{NaLi}**	*[converged to* ***A6^{NaLi}*** *]*				
	**A6^{NaLi}**	-710.0180507	0.034718	-0.001635	–	-710.2353525
	**A7^{NaLi}**	-710.0120046	0.034401	-0.002207	–	-710.2299860
M^1 || M^2 || Na	**A1a^{NaNa}**	-589.7137285	0.036533	0.002425	–	-589.8689215
	**A1b^{NaNa}**	*[same as* ***A1a^{NaNa}*** *]*				
	**A1c^{NaNa}**	-589.7132047	0.036391	0.000467	–	-589.8698674
	**TS$_{A1/A2}^{NaNa}$**	-589.6364304	0.032117	-0.000861	-291.8639	-589.8102322

	**A2^{NaNa}**	-589.6364963	0.034047	0.000238	–	-589.8089413
	**A3^{NaNa}**	-864.7694235	0.035537	-0.004941	–	-864.9767923
	**TS$_{A3/A4}^{NaNa}$**	-864.7028099	0.035990	0.000139	-279.9431	-864.9409101
	**A4^{NaNa}**	-864.7085563	0.037624	0.001929	–	-864.9575898
	**TS$_{A4/A5}^{NaNa}$**	-864.6741050	0.031392	-0.005087	-554.5486	-864.9278390
	**A5^{NaNa}**	-864.7647072	0.033025	-0.003619	–	-864.9816737
	**A6^{NaNa}**	-864.7665648	0.032927	-0.005231	–	-864.9885194
	**A7^{NaNa}**	*[same as **A6^{NaNa}**]*				
M[1] || Na M[2] || K	**A1a^{NaK}**	-455.7321640	0.035738	0.000403	–	-455.8949361
	**A1b^{NaK}**	-455.7293946	0.035636	0.000138	–	-455.8942847
	**A1c^{NaK}**	*[converged to **A1b^{NaK}**]*				
	**TS$_{A1/A2}^{NaK}$**	-455.6573247	0.030218	-0.004574	-971.5181	-455.8412397
	**A2^{NaK}**	-455.6589172	0.034041	-0.001492	–	-455.8363856
	**A3^{NaK}**	-730.7913339	0.034711	-0.007813	–	-731.0009480
	**TS$_{A3/A4}^{NaK}$**	-730.7262428	0.035527	-0.001335	-271.1105	-730.9694661
	**A4^{NaK}**	-730.7302664	0.037058	0.000394	–	-730.9848906
	**TS$_{A4/A5}^{NaK}$**	-730.6915735	0.030551	-0.006960	-545.8906	-730.9538488
	**A5^{NaK}**	-730.7883469	0.032200	-0.005617	–	-731.0094842
	**A6^{NaK}**	-730.7858673	0.031938	-0.007993	–	-731.0133829

	**A7**^{NaK}	-730.7906942	0.032201	-0.007228	–	-731.0161359
M[1] ‖ Na M[2] ‖ Rb	**A1a**^{NaRb}	-451.5074971	0.035317	-0.001598	–	-451.6722331
	**A1b**^{NaRb}	-451.5037668	0.035218	-0.001713	–	-451.6711726
	**A1c**^{NaRb}	*[converged to* ***A1b***^{NaRb} *]*				
	**TS**$_{A1/A2}^{NaRb}$	-451.4329358	0.029864	-0.006330	-1035.2537	-451.6200890
	**A2**^{NaRb}	-451.4354244	0.033892	-0.003024	–	-451.6139346
	**A3**^{NaRb}	-726.5682121	0.034445	-0.009628	–	-726.7785990
	**TS**$_{A3/A4}^{NaRb}$	-726.5030722	0.035299	-0.002757	-270.9883	-726.7474280
	**A4**^{NaRb}	-726.5067973	0.036810	-0.001056	–	-726.7626293
	**TS**$_{A4/A5}^{NaRb}$	-726.4669569	0.030265	-0.008449	-548.4702	-726.7306340
	**A5**^{NaRb}	-726.5649990	0.031844	-0.007217	–	-726.7873776
	**A6**^{NaRb}	-726.5613024	0.031540	-0.009919	–	-726.7905020
	**A7**^{NaRb}	-726.5681496	0.031904	-0.008808	–	-726.7941132
M[1] ‖ Na M[2] ‖ Cs	**A1a**^{NaCs}	-447.5542160	0.035078	-0.003747	–	-447.7220162
	**A1b**^{NaCs}	-447.5489289	0.034915	-0.003223	–	-447.7203569
	**A1c**^{NaCs}	*[converged to* ***A1b***^{NaCs} *]*				
	**TS**$_{A1/A2}^{NaCs}$	-447.4797084	0.029740	-0.007148	-1035.8233	-447.6711612
	**A2**^{NaCs}	-447.4831418	0.033591	-0.005651	–	-447.6638568
	**A3**^{NaCs}	-722.6163335	0.034331	-0.010815	–	-722.8280412

	$\mathbf{TS_{A3/A4}^{NaCs}}$	-722.5509606	0.035188	-0.003742	-267.2224	-722.7977343
	$\mathbf{A4^{NaCs}}$	-722.5542306	0.036675	-0.002066	–	-722.8126082
	$\mathbf{TS_{A4/A5}^{NaCs}}$	-722.5120395	0.029952	-0.009733	-533.4842	-722.7798739
	$\mathbf{A5^{NaCs}}$	-722.6121322	0.031550	-0.008470	–	-722.8375080
	$\mathbf{A6^{NaCs}}$	-722.6076179	0.031211	-0.011652	–	-722.8398917
	$\mathbf{A7^{NaCs}}$	-722.6161342	0.031675	-0.009866	–	-722.8440006
M^1 $\parallel$ Li M^2 $\parallel$ Na	$\mathbf{A1a^{LiNa}}$	*[converged to **A1c**^{NaLi}]*				
	$\mathbf{A1b^{LiNa}}$	*[converged to **A1c**^{NaLi}]*				
	$\mathbf{A1c^{LiNa}}$	*[same as **A1c**^{NaLi}]*				
	$\mathbf{TS_{A1/A2}^{LiNa}}$	-434.8824234	0.033942	0.002391	-108.9436	-435.0512437
	$\mathbf{A2^{LiNa}}$	-434.8824245	0.034727	0.002192	–	-435.0508839
	$\mathbf{A3^{LiNa}}$	-555.2647995	0.038070	-0.000295	–	-555.4657057
	$\mathbf{TS_{A3/A4}^{LiNa}}$	-555.1978222	0.038690	0.005469	-320.6329	-555.4237073
	$\mathbf{A4^{LiNa}}$	-555.2139932	0.040890	0.006827	–	-555.4514933
	$\mathbf{TS_{A4/A5}^{LiNa}}$	-555.1833366	0.035012	0.001769	-727.5491	-555.4278136
	$\mathbf{A5^{LiNa}}$	-555.2571402	0.035830	0.002113	–	-555.4623720
	$\mathbf{A6^{LiNa}}$	-555.2647169	0.035962	0.000972	–	-555.4743934
	$\mathbf{A7^{LiNa}}$	-555.2700891	0.036239	0.001444	–	-555.4790826
	$\mathbf{A1^{LiLi}}$	-280.2302593	0.04025	0.009974	–	-280.3675166

M^1 ‖ M^2 ‖ Li	**TS$_{A1/A2}^{LiLi}$**	*[not converged]*				
	**A2^{LiLi}**	*[converged to **A1^{LiLi}**]*				
	**A3^{LiLi}**	-400.5136969	0.039886	0.004776	–	-400.7071458
	**TS$_{A3/A4}^{LiLi}$**	-400.4484275	0.040172	0.008409	-326.7331	-400.6675897
	**A4^{LiLi}**	-400.4762605	0.043006	0.010540	–	-400.7051398
	**TS$_{A4/A5}^{LiLi}$**	-400.4415928	0.038207	0.006787	-923.8314	-400.6748322
	**A5^{LiLi}**	*[converged to **A6^{LiLi}**]*				
	**A6^{LiLi}**	-400.5121683	0.037585	0.004396		-400.7192607
M^1 ‖ M^2 ‖ K	**A1^{KK}**	-321.7431302	0.034742	-0.001995		-321.9192147
	**TS$_{A1/A2}^{KK}$**	-321.6781609	0.029549	-0.006715	-1004.6677	-321.8657285
	**A2^{KK}**	-321.6797595	0.033424	-0.003594		-321.8597377
	**A3^{KK}**	-462.8314587	0.033522	-0.011272		-463.0493330
	**TS$_{A3/A4}^{KK}$**	-462.7662244	0.034314	-0.004634	-244.342	-463.0217201
	**A4^{KK}**	-462.7674979	0.035482	-0.003392		-463.0332097
	**TS$_{A4/A5}^{KK}$**	-462.7424861	0.030091	-0.009398	-769.3539	-463.0098768
	**A5^{KK}**	-462.8316587	0.030783	-0.009581		-463.0651148
	**A6^{KK}**	-462.8274820	0.030450	-0.010005		-463.0663830
	**A1^{RbRb}**	-313.2900351	0.033777	-0.006846		-313.4725569
	**TS$_{A1/A2}^{RbRb}$**	-313.2311345	0.029013	-0.009772	-1143.1442	-313.4221886

M[1] || M[2] || Rb	**A2**^{RbRb}	-313.2343534	0.033326	-0.006226		-313.4131560
	**A3**^{RbRb}	-450.1599022	0.032691	-0.016439		-450.3795319
	**TS**$_{A3/A4}^{RbRb}$	-450.0946557	0.033634	-0.008657	-182.5431	-450.3554718
	**A4**^{RbRb}	-450.0948766	0.034410	-0.008195		-450.3624154
	**TS**$_{A4/A5}^{RbRb}$	-450.0953951	0.030306	-0.010995	-866.4548	-450.3447214
	**A5**^{RbRb}	-450.1582001	0.029782	-0.014289		-450.3975881
	**A6**^{RbRb}	-450.1533228	0.029404	-0.014751		-450.3979034
M[1] || M[2] || Cs	**A1**^{CsCs}	-305.3771058	0.033205	-0.008485		-305.5706406
	**TS**$_{A1/A2}^{CsCs}$	-305.323320	0.028709	-0.011599	-1188.0048	-305.5222453
	**A2**^{CsCs}	-305.3281165	0.032769	-0.008122		-305.5102364
	**A3**^{CsCs}	-438.2971723	0.032151	-0.019945		-438.5237391
	**TS**$_{A3/A4}^{CsCs}$	-438.2284075	0.032157	-0.013681	-228.6542	-438.4878661
	**A4**^{CsCs}	-438.2393262	0.033918	-0.011387		-438.5115539
	**TS**$_{A4/A5}^{CsCs}$	-438.2314272	0.029638	-0.014318	-881.803	-438.4930072
	**A5**^{CsCs}	*[converged to **A6**^{CsCs}]*				
	**A6**^{CsCs}	-438.2934399	0.028988	-0.017909		-438.5460806

[a]BS1: B3LYP / SDD (Li, Na, K, Rb, Cs), 6-31G* (C, H, O)
[b]BS2: B3LYP-D / SDD (Li, Na, K, Rb, Cs), 6-311++G** (C, H, O) / PCM (ε = 26.0 for molten salt)

Supplementary Table 11. The DFT-optimized Geometries (in XYZ file format)

Li[formate]

C	-0.00006700	-0.57379400	-0.00012500
O	1.11676300	0.03038900	0.00006400
O	-1.11657800	0.03002500	0.00007600
H	0.00057700	-1.67993100	0.00001800
Li	-0.00055200	1.54646100	-0.00012700

Na[formate]

C	-1.03441200	-0.00005600	0.00001600
O	-0.45777700	-1.12710200	-0.00001100
O	-0.45796100	1.12706000	-0.00001100
H	-2.14489400	-0.00006000	0.00002900
Na	1.42520700	0.00006600	0.00000500

K[formate]

C	-1.48432100	0.00006900	-0.00022700
O	-0.92323300	-1.13206900	0.00026500
O	-0.92312700	1.13206700	0.00026500
H	-2.60127400	-0.00005000	-0.00147000
K	1.38305700	-0.00001800	-0.00007400

Rb[formate]

C	-1.99483800	0.00001300	-0.00023700
O	-1.43779700	-1.13310700	0.00027100
O	-1.43765300	1.13313100	0.00027100
H	-3.11337600	0.00014100	-0.00154400
Rb	1.02935100	-0.00001100	-0.00003700

Cs[formate]

C	-2.35975000	0.00042800	0.00080300
O	-1.80654900	-1.13390000	-0.00081700
O	-1.80474000	1.13393600	-0.00081800
H	-3.48031400	0.00171200	0.00444800
Cs	0.84598400	-0.00008300	0.00006900

LiOH

O	0.00003000	-0.31529000	0.00000000
H	-0.00032800	-1.27027400	0.00000000
Li	0.00003000	1.26419800	0.00000000

NaOH

O	0.03701900	1.05545400	0.00000000
H	-0.70336100	1.67067200	0.00000000
Na	0.03701900	-0.91948200	0.00000000

KOH

O	0.02149000	1.55503400	0.00000000
H	-0.58021700	2.30796100	0.00000000
K	0.02149000	-0.77622300	0.00000000

RbOH

O	0.01731000	2.00109400	0.00000000
H	-0.77893500	2.55044200	0.00000000
Rb	0.01731000	-0.50160000	0.00000000

CsOH

O	0.01192300	2.26046000	0.00000000
H	-0.75115400	2.85540400	0.00000000
Cs	0.01192300	-0.38071100	0.00000000

LiH

Li	0.00000000	0.00000000	0.40517200
H	0.00000000	0.00000000	-1.21551700

NaH

Na	0.00000000	0.00000000	0.15683800
H	0.00000000	0.00000000	-1.72521800

KH

K	0.00000000	0.00000000	0.11568000
H	0.00000000	0.00000000	-2.19791900

RbH

Rb	0.00000000	0.00000000	0.06474400
H	0.00000000	0.00000000	-2.39554200

CsH

Cs	0.00000000	0.00000000	0.04732100
H	0.00000000	0.00000000	-2.60267900

Li_2[oxalate]

C	-0.00018000	-0.79723200	-0.00004300
O	-1.13039100	-1.35783200	-0.00004000
O	1.12983100	-1.35826100	-0.00010800
C	0.00018400	0.79722500	0.00004400
O	1.13039200	1.35784500	0.00004100
O	-1.12983600	1.35825000	0.00009700
Li	2.35313300	-0.00044500	-0.00004700
Li	-2.35313100	0.00045400	0.00007100

Na_2[oxalate]

C	-0.00002000	0.80703900	0.00001700
O	-1.12153400	1.38511500	-0.00040400
O	1.12153300	1.38532700	0.00015400
C	0.00000700	-0.80702000	0.00003700
O	1.12152500	-1.38508600	-0.00018800
O	-1.12154100	-1.38531900	0.00044800
Na	2.74172300	-0.00008500	0.00020000
Na	-2.74170300	0.00004800	-0.00023700

K_2[oxalate]

C	-0.00008500	0.81072600	0.00008700
O	-1.12287200	1.38508300	-0.00021200
O	1.12213900	1.38595000	-0.00003800
C	0.00008500	-0.81072600	0.00000300
O	1.12287200	-1.38508300	-0.00028600
O	-1.12213900	-1.38595000	-0.00024000
K	3.19348200	0.00001200	0.00023400
K	-3.19348200	-0.00001200	0.00006400

Rb_2[oxalate]

C	-0.00044700	0.81154200	0.00014300
O	-1.12309400	1.38538800	0.00056400
O	1.12207700	1.38555400	0.00024500
C	0.00044700	-0.81154200	0.00006100
O	1.12309400	-1.38538800	0.00048700
O	-1.12207700	-1.38555400	0.00004500
Rb	3.35886600	0.00009600	-0.00007200
Rb	-3.35886600	-0.00009600	-0.00025100

Cs_2[oxalate]

C	0.00003500	0.81139900	-0.00131900
O	-1.12422500	1.38184800	-0.00530600
O	1.12414000	1.38204900	0.00290800
C	0.00011900	-0.81146000	-0.00151900

O	1.12431400	-1.38194100	-0.00439100
O	-1.12406900	-1.38199100	0.00123700
Cs	3.53681700	0.00001100	0.00022900
Cs	-3.53685700	0.00000100	0.00088800

[NaLi][carbonite]

C	0.83533900	0.52911400	0.00010900
O	-0.16271300	1.28033100	-0.00002200
O	0.66752200	-0.81028400	0.00017800
Na	-1.48547200	-0.46885600	-0.00007200
Li	2.42989500	-0.59254500	-0.00037100

[NaNa][carbonite]

C	-0.19398600	0.64224900	0.00029300
O	0.87586000	1.28658000	-0.00005500
O	-0.22662200	-0.67743000	0.00045400
Na	1.97182500	-0.63067100	-0.00021100
Na	-2.33818800	-0.16266500	-0.00023900

[NaK][carbonite]

C	0.36836500	0.67846000	0.00005900
O	1.46371600	1.28036300	0.00001900
O	0.24246200	-0.61687700	0.00041000
Na	2.45954900	-0.70286400	-0.00018600
K	-2.25866600	-0.08669200	-0.00009100

[NaRb][carbonite]

C	1.05043900	0.67875100	0.00001700
O	2.14433200	1.28162200	0.00002400
O	0.90572800	-0.60716600	0.00029900
Na	3.12965600	-0.71633000	-0.00013800
Rb	-1.76025200	-0.04293300	-0.00003200

[NaCs][carbonite]

C	1.54687900	0.70134400	-0.00002700
O	2.65549500	1.27718100	0.00000500
O	1.35922600	-0.57434100	-0.00009800
Na	3.58407100	-0.75271400	0.00004600
Cs	-1.46952400	-0.02819900	0.00000700

[LiNa][carbonite]

C	0.36984700	-0.44022600	-0.00001800
O	1.60483800	-0.70932700	0.00003200
O	0.01647000	0.86191800	-0.00014700
Na	-1.88036300	-0.18046200	0.00009600
Li	1.83148200	1.13523500	-0.00000900

[LiLi][carbonite]

C	-0.04113000	-0.56553600	0.00001500
O	1.21118500	-0.43667300	-0.00001600
O	-0.76960200	0.60319500	0.00000400
Li	0.87709500	1.38861400	-0.00006600
Li	-1.97238700	-0.70160200	0.00006900

[KK][carbonite]

C	-0.14297300	0.79658000	0.00040500
O	0.82759600	1.56527600	-0.00012800
O	-0.16111500	-0.48406900	0.00074200
K	2.48839300	-0.47340900	-0.00018000
K	-2.72386700	-0.23338700	-0.00020600

[RbRb][carbonite]

C	-0.10408300	0.94021500	0.00012600
O	0.80923700	1.76689900	-0.00004300
O	-0.10705000	-0.32527500	0.00025000
Rb	2.72890800	-0.29147100	-0.00003100
Rb-	2.86385400	-0.17269900	-0.00003500

[CsCs][carbonite]

C	0.06515000	1.11131700	0.00038700
O	-0.83001200	1.94920300	-0.00019600
O	0.11340800	-0.14117500	0.00089100
Cs	-2.94412500	-0.23361400	-0.00006600
Cs	3.04125100	-0.15060700	-0.00007700

A1c^{NaLi} (= **A1^{LiNa}**)

C	1.72401000	0.14122400	0.00013300
O	1.09386200	1.24095000	-0.00023600
O	1.23589800	-1.01677900	0.00007800
H	2.83044400	0.21735300	0.00064500
Na	-0.93314100	-1.19619300	-0.00012600
O	-2.06264100	0.57827300	0.00012600
H	-3.00536200	0.75594900	0.00012300
Li	-0.68051400	1.63930900	0.00002400

A3^{NaLi}

C	-3.01524000	-0.45318300	-0.00039200
H	-4.09872400	-0.69853000	-0.00086500
C	0.99238100	-0.02021300	0.00041100
O	1.53813600	-1.20497000	0.00013900
O	1.71718000	1.04369500	0.00027600
Na	3.50830900	-0.24054900	-0.00052600
O	-2.71911400	0.76895500	-0.00053800
O	-2.23028300	-1.44811200	0.00040900
Na	-0.69279500	1.58017600	0.00030800
Li	-0.39404600	-1.49117600	0.00028800

TS$_{A3/A4}^{NaLi}$

C	-1.39398600	0.70362300	0.48527600
H	-1.32302100	1.64241200	1.08518400
C	0.48860900	0.20486200	0.46222700
O	1.33742000	1.15285900	0.13877700
O	0.80915200	-1.01664200	0.27860800
Na	2.90257300	-0.39102700	-0.06733500
O	-2.10120200	-0.23957200	0.97447000
O	-1.40004900	0.92821900	-0.88032700
Na	-1.31733900	-1.39733400	-0.66701100
Li	0.05171400	1.99324900	-0.92820900

A4^{NaLi}

C	1.01811200	0.67550500	-0.60452700
H	0.93754700	1.65604400	-1.15062300
C	-0.45502800	0.17127000	-0.34102200
O	-1.37208500	1.08246000	-0.10068000
O	-0.70545100	-1.06045700	-0.16789200
Na	-2.81830300	-0.55240800	0.08721000
O	1.82968500	-0.18984800	-1.15997300
O	1.27189300	0.97072500	0.83120900
Na	1.71596700	-1.20528200	0.70865400
Li	-0.12756800	2.05828800	0.94936600

TS$_{A4/A5}^{NaLi}$

C	-1.15894100	0.71383400	0.07459300

H	−0.88516100	1.08648600	1.58124500
C	0.36067800	0.20824100	0.00941600
O	1.27930700	1.10153400	0.09782300
O	0.67568100	−1.03688000	−0.09972900
Na	2.80028600	−0.42586500	−0.07135200
O	−2.06739600	−0.20185700	0.06797000
O	−1.27440700	1.86358700	−0.53504400
Na	−1.25705100	−2.10406200	0.04042500
Li	−0.06877100	2.46639400	0.66891000

A6^{NaLi}

C	−0.29704300	−0.88672700	0.00006200
H	3.98736200	1.13516200	0.00140800
C	−0.12222000	0.70318200	−0.00016300
O	1.07690300	1.13269800	−0.00033500
O	−1.14204500	1.42991100	0.00000000
O	−1.48388300	−1.31522400	−0.00020200
O	0.75283000	−1.57057700	0.00006700
Na	−2.94584800	0.23257600	−0.00006300
Na	2.69422800	−0.57918500	0.00020000
Li	2.55519800	2.12144600	0.00048400

A7^{NaLi}

C	−0.29704300	−0.88672700	0.00006200
H	3.98736200	1.13516200	0.00140800
C	−0.12222000	0.70318200	−0.00016300
O	1.07690300	1.13269800	−0.00033500
O	−1.14204500	1.42991100	0.00000000
O	−1.48388300	−1.31522400	−0.00020200
O	0.75283000	−1.57057700	0.00006700
Na	−2.94584800	0.23257600	−0.00006300
Na	2.69422800	−0.57918500	0.00020000
Li	2.55519800	2.12144600	0.00048400

A1a^{NaNa} (= A1b^{NaNa})

C	1.82794300	0.56771500	0.00028000
H	2.68600400	1.27254700	0.00096500
O	2.08700500	−0.65908700	0.00068600
O	0.66743200	1.08575000	−0.00084500
Na	−0.03217400	−1.35741300	−0.00072200
O	−2.04394100	−0.73086500	0.00058400
H	−2.88081700	−1.20163400	0.00039400
Na	−1.46390000	1.26254200	0.00013600

A1c^{NaNa}

C	−1.92476000	−0.07583300	0.00012100
O	−1.29222600	−1.16742600	−0.00011000
O	−1.45155000	1.09288300	0.00016700
H	−3.03372800	−0.15403200	0.00016900
Na	0.65832600	1.57280900	−0.00005400
O	2.13204400	0.08959100	−0.00005100
H	3.09080800	0.14905300	−0.00010200
Na	0.83125100	−1.54193800	−0.00002300

TS$_{A1/A2}$^{NaNa}

C	0.23078600	0.57693100	−0.01854800
O	0.38508000	−0.74616500	0.01482400
O	1.24235800	1.32518300	−0.01406700
H	−1.29655500	0.98137100	−0.06076700
Na	2.54772500	−0.42139600	0.01197800
O	−2.41833500	0.86280400	−0.06905600
H	−2.75927000	1.47165500	0.59815700
Na	−1.72969900	−1.16489400	−0.00104200

A2^{NaNa}

C	0.22998300	0.56537100	−0.02577000
O	0.41179600	−0.75569400	0.01315500
O	1.22657300	1.33253800	−0.02304800
H	−1.42084000	0.99242700	−0.07022200
Na	2.56863100	−0.38868400	0.02054300
O	−2.48517800	0.84561100	−0.06127600
H	−2.82186700	1.42283300	0.63608300
Na	−1.69251400	−1.17378200	−0.00616900

A3^{NaNa}

C	3.14708800	0.00193100	−0.00131800
H	4.26033400	0.00738900	−0.00236200
C	−1.13934900	−0.00119800	0.00173000
O	−1.77966700	1.12506600	0.00065200
O	−1.77691000	−1.12867800	0.00084900
Na	−3.66516600	−0.00375700	−0.00221900
O	2.60248500	−1.13577800	−0.00123600
O	2.59122100	1.13421700	−0.00039400
Na	0.50024300	−1.67151800	0.00131300
Na	0.49185000	1.67796600	0.00098900

TS$_{A3/A4}$^{NaNa}

C	−1.37043700	0.30708700	0.63853400
H	−1.27687500	1.15659600	1.37514400
C	0.52120100	−0.10530300	0.45175800
O	1.39310100	0.85496100	0.45454000
O	0.82596600	−1.25918800	−0.00992400
Na	2.93984000	−0.63381600	−0.09263200
O	−1.98890800	−0.73004300	1.06451500
O	−1.53813200	0.72095800	−0.65419100
Na	−1.33015200	−1.56245700	−0.82766900
Na	−0.07913600	2.28165500	−0.42119200

A4^{NaNa}

C	−1.03793600	0.29258100	0.69890900
H	−0.95598300	1.18632700	1.40363600
C	0.46087000	−0.12357800	0.36206200
O	1.39848100	0.77597900	0.40845000
O	0.69538400	−1.29111000	−0.10220100
Na	2.81763200	−0.80532800	−0.12997100
O	−1.78021300	−0.67839700	1.18325700
O	−1.38432800	0.75937600	−0.62674500
Na	−1.62482600	−1.44529600	−0.81563100
Na	−0.01246200	2.36633900	−0.38817500

TS$_{A4/A5}$^{NaNa}

C	−1.02978200	0.15795100	0.44097300
H	−0.91623000	1.92188300	1.53492100
C	0.50451700	−0.03455900	0.22107700
O	1.33660300	0.91694200	0.08568400
O	0.82807100	−1.26097500	−0.02203300
Na	2.91805600	−0.58890800	−0.03510900
O	−1.61260300	−0.63674500	1.22262600
O	−1.50330100	0.54991500	−0.71802200
Na	−1.45767200	−1.85342800	−0.60964900

Na -0.39877800 2.51367000 -0.26917500

A5^{NaNa}

C -0.73908300 -1.09819400 -0.09172000
H 2.94737300 0.49823300 1.28454100
C -0.06176400 0.26964500 -0.48921500
O 1.04623600 0.23617200 -1.08514400
O -0.59151100 1.35900600 -0.06115900
Na 1.77521100 1.88991800 0.29641900
O -2.00181500 -1.11337800 -0.02252600
O 0.06150100 -2.02246000 0.17653700
Na -2.69379600 0.87648000 0.31282300
Na 2.16789600 -1.23927600 0.31252300

A6^{NaNa} (= A7^{NaNa})

C 0.87843300 0.95576700 -0.00014700
H -3.93699700 0.34664500 0.00118300
C 0.28024100 -0.53192100 -0.00027100
O -0.98508800 -0.62489100 -0.00060000
O 1.07502700 -1.50683900 0.00011900
Na -2.97085200 -1.43877900 0.00058100
O 2.13785100 1.05626400 -0.00055200
O 0.05084500 1.89660500 0.00025500
Na 3.12067100 -0.82745900 -0.00015600
Na -2.08110300 1.40634400 0.00026200

A1a^{NaK}

C 1.99352800 -0.86724300 -0.00010000
H 2.63738800 -1.77492900 -0.00027200
O 2.57176000 0.24686000 -0.00000500
O 0.73832200 -1.05810000 0.00000400
Na 0.62598500 1.37815000 0.00013400
O -1.47338100 1.56327300 -0.00004200
H -1.96836600 2.38803700 -0.00082300
K -1.80050700 -0.87292400 0.00003000

A1b^{NaK}

C -1.96670600 0.76840800 -0.00353000
O -2.25289300 -0.44780900 0.00994000
O -0.80039700 1.27653400 -0.00498700
H -2.81217100 1.49417400 -0.01961600
Na 1.29437500 1.61056500 0.00390700
O 2.30492000 -0.20197300 -0.00008400
H 3.25328400 -0.36192300 0.00098400
K 0.16357700 -1.49857600 -0.00221700

TS$_{A1/A2}$^{NaK}

C 0.66935800 0.61670300 0.00015100
O 0.61345600 -0.69571400 0.00006900
O 1.77610000 1.22643000 0.00008800
H -0.61614200 1.27975200 0.00025700
Na 2.80699500 -0.69046000 -0.00021700
O -1.79112700 1.56285700 0.00027200
H -1.83341900 2.52566400 0.00036300
K -1.95952400 -0.87679800 -0.00013500

A2^{NaK}

C 0.65042200 0.60856400 -0.02675500
O 0.66126700 -0.70602200 0.00517200
O 1.72104100 1.27486900 -0.02241300
H -0.95749600 1.40951400 -0.06391000
Na 2.85807900 -0.59296300 0.02032400
O -1.98376800 1.58867800 -0.03615900
H -2.07564600 2.42982400 0.42735200
K -1.86824100 -0.95938500 0.00003800

A3^{NaK}

C 3.18969400 -0.39275500 -0.00003800
H 4.26514100 -0.10274400 0.00102500
C -1.28544900 -0.13663000 0.00146500
O -2.15172200 0.81850500 0.00178800
O -1.64841200 -1.38044500 -0.00039000
Na -3.73941300 -0.71250400 -0.00108700
O 2.93531300 -1.62386300 0.00299300
O 2.34408800 0.55029100 -0.00417400
Na 0.70318000 -1.42817400 -0.00083900
K 0.30914800 2.10055800 0.00052000

TS$_{A3/A4}$^{NaK}

C 1.15779100 0.62947300 0.69388100
H 1.40730200 -0.14801300 1.47794000
C -0.67600700 0.13460100 0.40714400
O -1.07733300 -1.07047400 0.59615300
O -1.39779500 0.99297400 -0.21304100
Na -3.08638100 -0.42189300 -0.10923800
O 1.18871900 1.84776700 1.11322600
O 1.60972700 0.31879700 -0.54648900
Na 0.42898500 2.22969300 -0.88596600
K 1.17614800 -2.15972300 -0.24924400

A4^{NaK}

C -0.15978900 -1.01556800 0.72541900
H -0.96208600 -0.73688900 1.48979000
C 0.52964000 0.38007600 0.33322600
O -0.09913400 1.49244200 0.51546800
O 1.65705600 0.34881000 -0.27899000
Na 1.72927700 2.51628400 -0.14195700
O 0.69824500 -1.90152800 1.20295500
O -0.75756900 -1.31227100 -0.52920500
Na 1.34459600 -1.94164400 -0.84784800
K -2.47675800 0.48469400 -0.22292700

TS$_{A4/A5}$^{NaK}

C -0.10428200 -0.97698800 0.50124400
H -1.66396700 -0.31163600 1.82884300
C 0.52101500 0.42310400 0.21217800
O -0.09732100 1.52895100 0.20013900
O 1.74918700 0.32620400 -0.19530200
Na 1.84280900 2.50464600 -0.04662300
O 0.54818000 -1.74631800 1.25721800
O -0.72269100 -1.34682600 -0.58529100
Na 1.57040700 -2.02315500 -0.67896000
K -2.64213900 0.43381300 -0.18642500

A5^{NaK}

C 0.79488800 -1.18466600 -0.14816500
H -2.56807600 1.73205700 1.19197300
C 0.42946900 0.31378200 -0.49208100
O -0.62164400 0.55001500 -1.13695400

O	1.16402300	1.24690600	0.00953300
Na	-0.92369000	2.40251300	0.18472400
O	2.02989900	-1.45458300	-0.03846000
O	-0.17842600	-1.94317100	0.03358400
Na	3.09404700	0.32719600	0.42434300
K	-2.51593700	-0.72246800	0.26358700

$A6^{NaK}$

C	-1.03106600	-0.97776600	-0.00014000
H	3.89495600	0.99357300	0.00144300
C	-0.62539900	0.57575500	-0.00043500
O	0.61343400	0.83432100	-0.00134100
O	-1.54130300	1.44375300	0.00035500
Na	2.27944300	2.18988900	0.00023900
O	-2.27320800	-1.23281700	-0.00038500
O	-0.09620500	-1.80495700	0.00031000
Na	-3.47637500	0.50989300	0.00044600
K	2.39938700	-1.16850100	0.00015600

$A7^{NaK}$

C	1.42084200	0.90137800	-0.00032800
H	-3.46116900	1.50816200	0.00138200
C	0.54460500	-0.44212300	-0.00026700
O	-0.71086200	-0.29586200	-0.00065000
O	1.14269800	-1.55464100	-0.00014400
O	2.67816800	0.76066200	-0.00038800
O	0.79083000	1.98367800	0.00003000
Na	3.27336600	-1.27455900	-0.00025800
Na	-1.40554000	1.90407300	0.00012800
K	-3.16233000	-0.96521300	0.00067600

$A1a^{NaRb}$

C	-2.31430900	-1.09742300	-0.00006100
H	-2.78161500	-2.10818800	-0.00015200
O	-3.08908400	-0.10902800	-0.00030500
O	-1.04599400	-1.05578900	0.00028300
Na	-1.37214800	1.34908100	0.00006300
O	0.63061400	1.99700500	-0.00002600
H	0.91665700	2.91593800	0.00004700
Rb	1.59135500	-0.42488100	0.00000400

$A1b^{NaRb}$

C	-2.33039800	0.40692500	-0.00003500
O	-2.18991400	-0.83396500	0.00007500
O	-1.40840900	1.28299300	-0.00000100
H	-3.37320800	0.80330800	-0.00012000
Na	0.44039500	2.31562500	-0.00000700
O	2.08613000	1.05743100	0.00002300
H	3.01657000	1.30333300	0.00010600
Rb	0.58357400	-1.13707400	-0.00001300

$TS_{A1/A2}^{NaRb}$

C	1.28846700	0.64866400	0.00008800
O	1.04049300	-0.63694500	0.00021300
O	2.47113600	1.09616800	-0.00008000
H	0.15093700	1.48745200	0.00008900
Na	3.21409900	-0.94879900	-0.00007000
O	-0.96969800	2.00483800	0.00003600
H	-0.80659000	2.95520000	-0.00016600
Rb	-1.69637000	-0.47595500	-0.00002800

$A2^{NaRb}$

C	1.25781100	0.65313000	-0.02946500
O	1.05705700	-0.64078600	0.00041000
O	2.41738400	1.14890000	-0.02083400
H	-0.20067500	1.73450000	-0.06839400
Na	3.24856000	-0.87693500	0.02169900
O	-1.15933800	2.12475100	-0.03342400
H	-1.05521300	2.97550400	0.40999100
Rb	-1.63637700	-0.54176800	0.00073800

$A3^{NaRb}$

C	-3.07832600	1.09609700	0.00050000
H	-4.17504800	0.89887700	0.00096800
C	1.41028800	0.42280300	-0.00056800
O	2.25750500	-0.54430700	0.00020700
O	1.79382100	1.66242500	-0.00040200
Na	3.87319200	0.95900300	0.00065500
O	-2.71882600	2.30057900	0.00120500
O	-2.31612200	0.08389900	-0.00069300
Na	-0.51872100	1.83116900	-0.00109000
Rb	-0.40126800	-1.85743100	0.00004600

$TS_{A3/A4}^{NaRb}$

C	0.47452400	-1.32974300	0.72851800
H	-0.28210500	-1.14330100	1.54829600
C	0.96279500	0.49440500	0.40912700
O	0.15934300	1.45564500	0.67471700
O	2.02523300	0.67312500	-0.28594800
Na	1.70203600	2.84976800	-0.12695000
O	1.53182300	-1.96827600	1.10436300
O	-0.08172600	-1.57765100	-0.48103100
Na	2.14032500	-1.52980000	-0.93502000
Rb	-2.15365300	0.08035000	-0.12944000

$A4^{NaRb}$

C	0.52833600	-1.04070500	0.75143200
H	-0.27223100	-0.88756700	1.55000900
C	0.96887600	0.44917100	0.33524900
O	0.19727600	1.45138800	0.57857300
O	2.04875200	0.59118100	-0.34577200
Na	1.81262800	2.74291000	-0.16024600
O	1.54424700	-1.76626100	1.19539500
O	-0.06400500	-1.44749500	-0.46834200
Na	2.09771000	-1.71636200	-0.88319100
Rb	-2.20364700	0.06795300	-0.11543600

$TS_{A4/A5}^{NaRb}$

C	0.55505000	-1.00420300	0.52638200
H	-1.02430600	-0.52941600	1.91598800
C	1.00248800	0.45895900	0.22353100
O	0.27779100	1.49462500	0.27544700
O	2.20980700	0.49518100	-0.25706100
Na	2.08883000	2.66635100	-0.06906500
O	1.31950000	-1.68478100	1.26428500
O	-0.05233800	-1.45273100	-0.53274500
Na	2.29132500	-1.85260200	-0.71555400
Rb	-2.33893800	0.10895400	-0.10227200

$A5^{NaRb}$

C	-1.15375500	-1.21072700	0.18579200

H	1.84798800	2.27686200	-1.09734300
C	-0.98571800	0.32867600	0.50352500
O	0.00220400	0.70784100	1.17782900
O	-1.81432100	1.15311300	-0.04305600
Na	0.08145000	2.59007500	-0.14043100
O	-2.34412600	-1.62910100	0.03870700
O	-0.09254800	-1.85168100	0.06108200
Na	-3.59926300	-0.00223200	-0.50035600
Rb	2.26149000	-0.33762700	-0.15855100

A6^{NaRb}

C	-1.36161000	-0.99585600	-0.00025800
H	3.18405500	1.98932100	0.00197200
C	-1.20294000	0.60159900	-0.00057200
O	-0.02102300	1.05087400	-0.00151700
O	-2.24375300	1.31751100	0.00017100
Na	1.32065600	2.72708000	0.00071700
O	-2.55067200	-1.44071300	-0.00024400
O	-0.31065100	-1.66675900	0.00000900
Na	-4.00792500	0.09346000	0.00043100
Rb	2.23708100	-0.66856800	0.00008200

A7^{NaRb}

C	-2.14524700	0.83376700	-0.00019800
H	2.61305700	2.14371000	0.00045800
C	-1.09993600	-0.38436400	0.00040900
O	0.12446500	-0.07625400	0.00073300
O	-1.54879100	-1.56652300	0.00028300
O	-3.37423300	0.53086100	-0.00028000
O	-1.66289700	1.98941800	-0.00033700
Na	-3.69548900	-1.56382700	-0.00013600
Na	0.53003400	2.18620700	0.00025100
Rb	2.79377600	-0.50557600	-0.00016700

A1a^{NaCs}

C	-2.64606600	-1.19162300	-0.00001700
H	-3.04524300	-2.23224700	-0.00016400
O	-3.48678000	-0.25816800	-0.00036200
O	-1.38378500	-1.06563900	0.00044100
Na	-1.84245900	1.29102600	0.00015800
Cs	1.40605400	-0.26536600	-0.00003600
O	0.07593700	2.15699300	-0.00002900
H	0.21275500	3.11034900	0.00008400

A1b^{NaCs}

C	-2.24554800	-1.35925200	-0.00004200
O	-1.26025500	-2.12412400	-0.00014200
O	-2.23362500	-0.08686800	0.00015000
H	-3.25909300	-1.82840000	-0.00038700
Na	-1.72072800	1.96115400	0.00008200
O	0.30151700	2.41157200	-0.00018400
H	0.67119300	3.30075000	-0.00034300
Cs	1.10051100	-0.29989400	0.00002700

TS$_{A1/A2}^{NaCs}$

C	-1.74537200	0.62530000	-0.00004400
O	-1.41441300	-0.63663400	-0.00012800
O	-2.95350600	1.00272400	-0.00008100
H	-0.69813200	1.53209400	0.00011200
Na	-3.56445000	-1.08583200	-0.00028100
O	0.37514200	2.19137800	0.00023900
H	0.07717500	3.10911700	0.00033000
Cs	1.49535200	-0.30742900	0.00004900

A2^{NaCs}

C	-1.71311900	0.63036100	0.00000300
O	-1.40389300	-0.63643300	0.00010000
O	-2.90754500	1.03738200	-0.00008000
H	-0.36152500	1.85598400	-0.00002800
Na	-3.57127200	-1.05411900	0.00000200
O	0.53392700	2.36828900	0.00003500
H	0.27755300	3.29822600	0.00011400
Cs	1.45212300	-0.35445400	-0.00001000

A3^{NaCs}

C	-2.67348300	2.05554700	0.00939900
H	-3.78825200	2.08260900	-0.00209800
C	1.62210300	0.46852700	-0.03369700
O	2.30414400	-0.61838900	-0.03637000
O	2.19661400	1.63280800	0.00794700
Na	4.13675500	0.61075200	0.03001300
O	-2.07874900	3.15928100	-0.07968100
O	-2.13098000	0.91489200	0.11088000
Na	-0.03760600	2.19525900	0.00883000
Cs	-0.67858800	-1.61458000	-0.00548300

TS$_{A3/A4}^{NaCs}$

C	0.90794400	-1.31769400	0.73754700
H	0.16155800	-1.12923100	1.56612100
C	1.36979200	0.50191700	0.39608700
O	0.58064800	1.45641100	0.70796300
O	2.40534600	0.68249500	-0.33847700
Na	2.09659800	2.85858300	-0.13707400
O	1.98100300	-1.93627200	1.11230400
O	0.33969900	-1.59116200	-0.45663900
Na	2.54315600	-1.52726500	-0.94897000
Cs	-1.95125100	0.04523000	-0.08404800

A4^{NaCs}

C	0.94359600	-1.04013200	0.75472500
H	0.15756500	-0.87415900	1.56407400
C	1.38612400	0.45083800	0.32696100
O	0.63953600	1.45885900	0.60635800
O	2.44454300	0.58073200	-0.39122000
Na	2.25160800	2.73372400	-0.16963800
O	1.96925500	-1.75703800	1.19913600
O	0.33870900	-1.45799100	-0.44578400
Na	2.49048500	-1.72412500	-0.89080400
Cs	-1.98973200	0.04923300	-0.07522300

TS$_{A4/A5}^{NaCs}$

C	1.02148800	-1.00045400	0.54501900
H	-0.55568400	-0.52552000	1.99894600
C	1.44595900	0.46494100	0.22883600
O	0.73119000	1.50120800	0.33429000
O	2.62850300	0.50185400	-0.31445400
Na	2.52508100	2.66945900	-0.09085100
O	1.80985300	-1.66465800	1.27320300

O	0.38741200	-1.46608900	-0.48655900
Na	2.71689600	-1.84650700	-0.74399500
Cs	-2.11575300	0.06741100	-0.07110200

A5^{NaCs}

C	1.53306100	-1.23182300	-0.20218900
H	-1.31118100	2.60565300	1.03235800
C	1.42906400	0.31882300	-0.49709700
O	0.47023100	0.74578700	-1.18326800
O	2.28203100	1.10304700	0.07315600
Na	0.48548000	2.65366500	0.10382900
O	2.70543100	-1.69544300	-0.03514600
O	0.44816400	-1.83547600	-0.11569900
Na	4.01013300	-0.12415300	0.54126600
Cs	-2.05745800	-0.20901100	0.11190800

A6^{NaCs}

C	-1.69820500	-1.03212000	-0.00040100
H	2.53595000	2.60733700	0.00198900
C	-1.66250700	0.57280400	-0.00055700
O	-0.51878500	1.10940400	-0.00152200
O	-2.75505100	1.21014800	0.00027000
Na	0.57153800	2.96150800	0.00062100
O	-2.85090800	-1.56808600	-0.00032700
O	-0.59886600	-1.61706900	-0.00011000
Na	-4.41607900	-0.14662800	0.00048700
Cs	2.06740300	-0.43436900	0.00009300

A7^{NaCs}

C	2.68792300	0.78430600	0.00022400
H	-1.95881700	2.49063400	0.00013000
C	1.56531900	-0.36337500	-0.00044600
O	0.36383400	0.02071800	-0.00093900
O	1.93766000	-1.57366100	-0.00026200
O	3.89599400	0.40364100	0.00033000
O	2.28164900	1.96823300	0.00036300
Na	4.07729800	-1.70494600	0.00016300
Na	0.10071400	2.30537900	-0.00027800
Cs	-2.49730700	-0.33040800	0.00011900

TS$_{A1/A2}^{LiNa}$

C	0.75908500	0.39940200	-0.01733000
O	0.70902400	-0.95341800	0.01962400
O	1.91950000	0.91426900	-0.00719600
H	-0.70652000	1.05126900	-0.06395500
O	-1.81749300	1.09536200	-0.07354000
H	-2.07278300	1.75753600	0.58030600
Na	-1.44302300	-1.01651200	0.00063000
Li	2.53659900	-0.82443000	0.02319600

A2^{LiNa}

C	0.75737800	0.40263500	-0.01910100
O	0.72361300	-0.95240600	0.02120700
O	1.91261600	0.92910800	-0.00932100
H	-0.75336600	1.06030800	-0.06732800
O	-1.84536500	1.08561800	-0.07193300
H	-2.10476900	1.73701700	0.59122900
Na	-1.42332400	-1.02750700	-0.00131200
Li	2.54784100	-0.80303800	0.02850300

A3^{LiNa}

C	2.60585500	-0.43725500	0.00033400
H	3.66288500	-0.77434200	0.00089100
C	-1.56791100	0.04136500	-0.00001500
O	-1.92845100	-1.24565000	-0.00012000
O	-2.52002000	0.90196300	0.00012500
O	2.40796900	0.80343100	0.00045800
O	1.73268500	-1.35640800	-0.00046600
Na	0.34275800	1.49499900	-0.00014700
Li	-0.08479400	-1.46091800	-0.00010900
Li	-3.64799100	-0.57975000	-0.00027900

TS$_{A3/A4}^{LiNa}$

C	1.17768100	0.36651800	-0.46596600
H	1.00371600	0.99539700	-1.37310900
C	-0.47572800	-0.66314800	-0.38369700
O	-1.69135900	-0.18627100	-0.52742200
O	-0.35874100	-1.70152200	0.39969600
O	2.14947900	-0.46960400	-0.50607800
O	0.90976900	0.97414300	0.72830600
Na	-0.97672500	1.82953100	0.13786600
Li	1.34779100	-0.97959600	1.09949400
Li	-2.19600600	-1.77854800	0.30002000

A4^{LiNa}

C	-0.85543700	0.14085400	-0.46538200
H	-0.71746100	-0.31909400	-1.49043800
C	0.56765700	0.66851100	-0.08735400
O	1.59462700	-0.14256400	-0.27958500
O	0.78247800	1.80735200	0.40496900
O	-1.81905600	1.06241600	-0.43312100
O	-1.08783000	-0.94316500	0.48232200
Na	0.59767900	-2.09142500	-0.00292400
Li	-2.52887500	0.06792600	0.85360900
Li	2.56484900	1.33083000	0.29383700

TS$_{A4/A5}^{LiNa}$

C	-0.05928200	-0.94065600	0.00251400
H	-0.46392000	-0.51012300	1.42074600
C	0.50975100	0.51330300	-0.06648600
O	-0.23725800	1.54950000	-0.22079700
O	1.79979000	0.71647100	0.06234300
O	0.87664500	-1.84866200	0.14240500
O	-1.14073600	-1.12931100	-0.67903900
Na	-2.17189800	0.39758800	0.38001100
Li	1.34800400	2.49986900	-0.18975300
Li	2.40681400	-1.03427000	0.30431000

A5^{LiNa}

C	-0.74119000	-0.83759800	0.12570500
H	2.25795300	1.20380100	-1.48677200
C	-0.25945900	0.63531900	0.37245900
O	0.83847200	0.86219400	0.93386600
O	-0.93254400	1.56927800	-0.21133700
O	-1.98530500	-0.96008700	-0.10360200
O	0.15749600	-1.69711000	0.07977800
Na	2.13323600	-0.61140300	-0.33432900
Li	1.09105100	2.14809300	-0.54313300
Li	-2.53925500	0.69894100	-0.59494400

A6^{LiNa}

C	-0.74635400	-0.79547100	-0.00012700
H	3.65573700	0.87718200	0.00045000
C	-0.45517600	0.77082200	-0.00001000
O	0.75766900	1.13155100	-0.00007500
O	-1.45017500	1.54229400	-0.00034400
O	-1.97842200	-1.10105100	-0.00029000
O	0.23063000	-1.56965900	0.00013100
Na	2.26037900	-0.73197800	0.00033000
Li	2.32663900	1.99185400	0.00089000
Li	-2.92275400	0.44060900	-0.00043700

$A7^{LiNa}$

C	-1.24452000	0.64607200	0.00016100
H	2.99331200	1.74380700	-0.00135600
C	-0.27021800	-0.60610000	0.00048400
O	0.96710700	-0.34433700	0.00077500
O	-0.79403100	-1.75248600	0.00013200
Na	3.13577300	-0.27551000	-0.00041700
O	-2.48078600	0.37308600	-0.00030700
O	-0.70452300	1.77493100	-0.00046000
Li	1.17446600	1.65040100	0.00026400
Li	-2.60797300	-1.43792600	0.00005400

$A1^{LiLi}$

C	-1.45539200	0.00008400	0.00007600
O	-0.89132400	-1.13047900	-0.00007000
O	-0.89117100	1.13039100	0.00033400
H	-2.56195700	0.00002400	0.00002700
O	2.11024300	0.00035400	-0.00013800
H	3.06910400	-0.00242700	-0.00007400
Li	0.93381000	-1.30768500	-0.00021700
Li	0.93393300	1.30760800	-0.00025400

$A3^{LiLi}$

C	2.39798300	-0.00013600	0.12580800
H	3.49397600	-0.00025700	0.28948200
C	-1.30418000	0.00021000	-0.32742900
O	-1.90071900	-1.11876500	-0.00729900
O	-1.90036300	1.11894400	-0.00594400
O	1.84932300	1.13529900	0.04403100
O	1.84878400	-1.13547000	0.04595700
Li	0.06870000	-1.45959400	-0.30174600
Li	0.06884800	1.45961900	-0.30031400
Li	-3.21521000	-0.00010600	0.70415400

$TS_{A3/A4}^{LiLi}$

C	1.19201900	0.32317600	-0.44360200
H	1.26089800	0.98035400	-1.33317600
C	-0.68279200	-0.18442100	-0.43413700
O	-1.64829500	0.72734200	-0.41636100
O	-0.92515300	-1.25977200	0.25593900
O	1.89843200	-0.74363200	-0.42779500
O	0.99026900	0.97607700	0.75424300
Li	-0.48600200	1.95664600	0.40177000
Li	-2.67905000	-0.65988600	0.28186100
Li	0.88562200	-1.10109300	1.07350200

$A4^{LiLi}$

C	1.19201900	0.32317600	-0.44360200
H	1.26089800	0.98035400	-1.33317600
C	-0.68279200	-0.18442100	-0.43413700
O	-1.64829500	0.72734200	-0.41636100
O	-0.92515300	-1.25977200	0.25593900
O	1.89843200	-0.74363200	-0.42779500
O	0.99026900	0.97607700	0.75424300
Li	-0.48600200	1.95664600	0.40177000
Li	-2.67905000	-0.65988600	0.28186100
Li	0.88562200	-1.10109300	1.07350200

$TS_{A4/A5}^{LiLi}$

C	0.92967400	-0.00001500	0.13271200
H	0.40525700	0.00047400	1.39107400
C	-0.55521400	0.00005100	0.05100400
O	-1.25047900	-1.12671500	-0.00181700
O	-1.25034600	1.12690100	-0.00221100
O	1.52046300	1.16255000	-0.07159800
O	1.52018600	-1.16272700	-0.07132300
Li	0.19154100	-2.31473000	-0.17305900
Li	0.19167400	2.31442400	-0.17230900
Li	-2.70674900	0.00005400	-0.09389000

$A6^{LiLi}$

C	0.63950200	0.73045400	-0.00019700
H	-3.68939200	0.86391900	0.00114000
C	-0.09334600	-0.67727500	-0.00048500
O	-1.36289200	-0.64124000	-0.00069100
O	0.62031700	-1.70907400	-0.00013400
O	1.90237900	0.67609100	0.00072500
O	-0.09219600	1.74586000	-0.00037700
Li	2.36226700	-1.08280800	0.00048700
Li	-1.90963100	1.30032800	-0.00004800
Li	-3.16210500	-0.80288300	0.00181500

$A1^{KK}$

C	-1.90770100	-1.26709700	0.00103700
O	-2.63167400	-0.24577700	0.00754300
O	-0.63852400	-1.30453200	-0.00831200
H	-2.42714500	-2.25628900	0.00348100
O	1.83006500	1.39994100	0.00335500
H	2.50771700	2.08485600	0.00559700
K	-0.67321100	1.53498600	-0.00291100
K	1.87777500	-1.06251500	0.00101700

$TS_{A1/A2}^{KK}$

C	0.16207000	0.75407100	0.00010800
O	0.24328500	-0.54964400	-0.00006300
O	1.16506000	1.51217300	0.00016200
H	-1.21565000	1.27750600	0.00023800
O	-2.39929600	1.43082700	0.00030300
H	-2.53806900	2.38504200	0.00038100
K	-2.27957900	-1.01741800	-0.00003000
K	2.84320500	-0.42120500	-0.00020600

$A2^{KK}$

C	0.16155800	0.72868000	-0.02408300
O	0.27430000	-0.56810600	-0.00379700
O	1.12626900	1.52650400	-0.01178600
H	-1.54961700	1.38379200	-0.06920200

O	−2.58352700	1.45366100	−0.04249500
H	−2.76180400	2.27907400	0.42554600
K	−2.21111900	−1.07534300	0.00414800
K	2.88510500	−0.36315300	0.00915600

A3^{KK}

C	3.72332000	0.03690200	−0.00201500
H	4.72088000	0.54202600	−0.00409300
C	−1.12784200	0.02512300	0.00098800
O	−1.86621100	1.07566800	0.00013800
O	−1.56607300	−1.17663100	0.00084600
O	3.72706800	−1.21605500	−0.00360200
O	2.71118200	0.80108200	0.00165100
K	0.58620900	2.19682300	0.00038700
K	−4.04722800	−0.36565200	−0.00141400
K	1.12725800	−1.66204900	0.00197300

TS$_{A3/A4}^{KK}$

C	−1.33056000	0.29144600	0.89124700
H	−1.09917200	1.18686500	1.55993700
C	0.43476700	−0.08436500	0.40559400
O	1.26132500	0.88985800	0.41406200
O	0.69386600	−1.24107500	−0.04050000
O	−1.82539700	−0.71587900	1.52987300
O	−1.81607900	0.66069600	−0.32558000
K	−0.39302200	2.73730000	−0.41735300
K	−1.82450900	−1.94950300	−0.66327100
K	3.26828000	−0.74454200	−0.07536700

A4^{KK}

C	−1.11108000	0.25285200	0.88951000
H	−0.93779200	1.17043700	1.56127700
C	0.41746000	−0.07118300	0.34875000
O	1.27208400	0.88867100	0.33886100
O	0.66809800	−1.22568500	−0.12285700
O	−1.62568000	−0.75051000	1.56731500
O	−1.73135300	0.64280400	−0.30695400
K	−0.42875800	2.77707900	−0.37608600
K	3.21125200	−0.78458300	−0.09635000
K	−1.91753000	−1.92421600	−0.62239300

TS$_{A4/A5}^{KK}$

C	0.96482500	−0.13850100	0.70677000
H	0.50794900	−1.59926100	1.76310500
C	−0.45287100	0.06192000	0.03386700
O	−1.23486500	−0.89930900	−0.25589100
O	−0.71184100	1.28001100	−0.28688300
O	1.29840900	0.72571700	1.57348100
O	1.77743800	−0.60313600	−0.21972900
K	0.58548800	−2.89189900	−0.22826300
K	−3.19804300	0.71424200	0.03528200
K	1.94872300	2.07410400	−0.47516400

A5^{KK}

C	−0.75468400	−1.37687000	0.11158400
H	3.50101100	0.89936900	−1.08144200
C	−0.22242200	0.06280000	0.48570300
O	0.85317600	0.14032500	1.14027900
O	−0.85960700	1.08079900	0.03802200
O	−2.00231200	−1.56269300	0.19003500
O	0.12211700	−2.19264300	−0.25997100
K	2.60565500	−1.36354000	−0.22333500
K	1.65654400	2.31104800	−0.12686700
K	−3.34353400	0.48716200	−0.24817800

A6^{KK}

C	1.03129000	1.17722300	−0.00011600
H	−4.32147900	0.02715600	0.00177500
C	0.44753500	−0.32366900	−0.00061000
O	−0.81415700	−0.43712200	−0.00201700
O	1.25749500	−1.28977700	0.00042300
O	2.28847900	1.29212600	−0.00040300
O	0.18958500	2.10620900	0.00054200
K	−2.35273600	1.72166800	−0.00015700
K	3.72184300	−0.73397700	0.00051700
K	−2.83872200	−1.96242600	0.00038900

A1^{RbRb}

C	−1.21542200	2.28709700	0.00365200
O	−2.36082700	1.78326400	0.03402500
O	−0.10433700	1.67555700	−0.03388900
H	−1.15673300	3.40513000	0.00994200
O	0.81973300	−2.01928600	0.01585200
H	1.10086100	−2.94196500	0.03322100
Rb	−1.60154100	−0.91773000	−0.00667500
Rb	2.15591600	0.22308100	0.00145900

TS$_{A1/A2}^{RbRb}$

C	0.12016900	1.02579400	−0.00002000
O	0.12160700	−0.27252200	0.00002300
O	1.15347100	1.73977600	−0.00007100
H	−1.17954600	1.62055300	−0.00000900
O	−2.37368200	1.90348300	0.00000000
H	−2.39419900	2.86813200	−0.00009100
Rb	−2.57438700	−0.67797500	0.00005500
Rb	2.88902400	−0.33849400	−0.00003800

A2^{RbRb}

C	0.11449300	1.00525500	−0.03755000
O	0.12432900	−0.28332800	−0.01180600
O	1.10730700	1.75969800	−0.02347400
H	−1.58913000	1.82246400	−0.09164200
O	−2.59571500	2.00091900	−0.03898400
H	−2.66311600	2.77973200	0.52900200
Rb	−2.52997500	−0.73467600	0.00206700
Rb	2.92127000	−0.30456800	0.00825800

A3^{RbRb}

C	4.14754900	−0.07042000	−0.00132100
H	5.13379000	0.46062200	−0.00265800
C	−0.91664000	0.03396900	−0.00049500
O	−1.60748700	1.10562200	−0.00001700
O	−1.36069800	−1.15691800	−0.00069400
O	4.18512200	−1.32200200	−0.00473800
O	3.11891700	0.67122100	0.00327500
Rb	0.97216200	2.30955900	0.00002700
Rb	1.43948300	−1.86230800	0.00115100
Rb	−4.01180900	−0.30198900	−0.00034100

TS$_{A3/A4}^{RbRb}$

C	−1.33020600	0.32082500	1.11518900

H	−1.04124200	1.21730600	1.76281900
C	0.32993600	−0.09241700	0.52247600
O	1.18130700	0.85458900	0.50617700
O	0.52189200	−1.25379100	0.06555500
O	−1.81089900	−0.66700100	1.80634500
O	−1.91624700	0.70774600	−0.05592900
Rb	−0.47674900	2.92586600	−0.29689800
Rb	−2.17226400	−2.02262800	−0.43434600
Rb	3.27697200	−0.89567300	−0.08405300

A4^{RbRb}

C	1.19974100	−0.26564300	1.09522700
H	0.96446500	−1.18048900	1.74846000
C	−0.33675900	0.07464300	0.47286300
O	−1.18364900	−0.88335800	0.44036200
O	−0.55310100	1.22937400	−0.00198400
O	1.65917500	0.73349100	1.81104700
O	1.88034000	−0.65132600	−0.05132300
Rb	0.56426600	−2.93222000	−0.27594600
Rb	−3.27621700	0.84577800	−0.08954400
Rb	2.15615400	2.05674100	−0.41131600

$TS_{A4/A5}^{RbRb}$

C	−0.22011400	−0.00042000	0.53478000
H	1.29810500	−0.00427100	1.13410000
C	0.10139400	−0.00918500	−1.04906100
O	1.30171600	0.00800700	−1.47270200
O	−0.94077000	−0.02893300	−1.76445700
O	−0.72565000	−1.14155000	0.93249200
O	−0.71367000	1.15296100	0.91528600
Rb	1.65026500	2.34325300	0.14964500
Rb	−3.06073400	0.01445300	0.05087900
Rb	1.62780000	−2.35397600	0.15262900

A5^{RbRb}

C	−0.80029500	−1.60910600	0.19358400
H	3.56582300	0.98511900	−1.00162800
C	−0.34131800	−0.13950000	0.53958700
O	0.73166300	0.00186600	1.18984700
O	−1.03860400	0.83301800	0.08470900
O	−2.02243100	−1.88227700	0.36700200
O	0.09822400	−2.36343300	−0.25162800
Rb	2.72415500	−1.49057800	−0.15933800
Rb	−3.64658800	0.10672900	−0.18097000
Rb	1.49359600	2.37825800	−0.05203900

A6^{RbRb}

C	−1.15135400	1.40217400	−0.00056000
H	4.31026300	−0.16564500	−0.00634400
C	−0.61436300	−0.11774000	0.00375900
O	0.64192100	−0.26896900	0.00913900
O	−1.45385300	−1.06038900	0.00168200
O	−2.40432200	1.55848400	−0.00045300
O	−0.27926500	2.30250600	−0.00337800
Rb	2.42071500	1.87211800	0.00002200
Rb	2.56359000	−2.15280500	−0.00118700
Rb	−4.05867800	−0.47050300	−0.00069400

A1^{CsCs}

C	0.84972800	2.64265900	0.02564000
O	2.05018500	2.39106400	0.27393000
O	−0.07126300	1.82323500	−0.27139100
H	0.54482300	3.72219900	0.06900300
O	−0.44582400	−2.09683000	0.13475600
H	−0.57219400	−3.04699100	0.25663700
Cs	1.95261700	−0.59798500	−0.03345900
Cs	−2.26781300	−0.01057700	0.00477100

$TS_{A1/A2}^{CsCs}$

C	−0.10144000	1.19676600	0.00008500
O	−0.06993200	−0.09442900	−0.00001400
O	−1.13958000	1.90282000	0.00005900
H	1.15497400	1.81864400	0.00023400
O	2.35046200	2.19098100	0.00035900
H	2.27856400	3.15393800	0.00042600
Cs	2.80100500	−0.51776000	0.00017000
Cs	−3.01832400	−0.28493300	−0.00025000

A2^{CsCs}

C	−0.10480500	1.20685800	0.00019000
O	−0.05294100	−0.06895000	0.00096500
O	−1.10099200	1.95063000	−0.00047900
H	1.59935700	2.09788400	−0.00015000
O	2.58926300	2.32601600	0.00013800
H	2.61403400	3.29132500	0.00038800
Cs	2.77374900	−0.57253300	−0.00008300
Cs	−3.04769800	−0.26913900	−0.00003200

A3^{CsCs}

C	4.44875400	−0.08738100	−0.05301800
H	5.40319400	0.50315500	−0.08674500
C	−0.82384900	0.01372300	0.00313600
O	−1.47021900	1.10304500	−0.00251600
O	−1.26160000	−1.17069500	0.00496100
O	4.56125500	−1.33232900	−0.11752100
O	3.38361200	0.59287200	0.04594900
Cs	1.20308000	2.47911700	0.00676800
Cs	1.66841700	−2.09227500	0.02151600
Cs	−4.12344300	−0.27055800	−0.01121000

$TS_{A3/A4}^{CsCs}$

C	0.20645200	0.00000000	−0.82015700
H	−0.95333200	0.00000000	−0.90930000
C	−0.02165900	0.00000000	1.31978100
O	−1.21454800	0.00000000	1.69338800
O	1.05625400	0.00000000	1.94075900
O	0.75092700	−1.14747500	−1.05678400
O	0.75092700	1.14747500	−1.05678400
Cs	3.25352600	0.00000000	−0.04209100
Cs	−1.72588900	−2.58985200	−0.10852800
Cs	−1.72589000	2.58985100	−0.10852800

A4^{CsCs}

C	0.00001500	−0.04398200	−0.66269300
H	0.00118700	1.04109000	−1.13463600
C	0.00029300	0.29203100	0.92207100
O	0.00163800	1.53377700	1.25471300

O	-0.00081800	-0.67080300	1.73923100
O	-1.17147100	-0.66599500	-0.99614800
O	1.17020800	-0.66849100	-0.99601900
Cs	-0.00323900	-3.06007500	0.01700300
Cs	-2.70725000	1.54412100	-0.08519700
Cs	2.71049800	1.53854900	-0.08518500

TS$_{A4/A5}$^{CsCs}

C	-0.22836000	-0.00004100	-0.49565700
H	1.29490000	0.00023800	-1.06303100
C	0.07751900	0.00017400	1.09423500
O	1.26671800	-0.00027900	1.54243700
O	-0.97753900	0.00068300	1.79285100
O	-0.72077200	1.14586800	-0.89346500
O	-0.72022700	-1.14632800	-0.89302200
Cs	-3.25442000	-0.00064000	-0.05039900
Cs	1.70684400	2.51839100	-0.11044300
Cs	1.70802500	-2.51776200	-0.11041000

A5^{CsCs} (=**A6^{CsCs}**)

C	-0.81096800	-1.74617300	0.17508100
H	3.75816500	1.09531900	-0.88822800
C	-0.40444200	-0.26052700	0.51399000
O	0.66367900	-0.07992600	1.16370200
O	-1.14815700	0.67907700	0.06782000
O	-2.01026700	-2.07178900	0.40871200
O	0.09633900	-2.46008100	-0.31808100
Cs	2.91027400	-1.62724800	-0.09787100
Cs	1.41605400	2.51998800	-0.03638300
Cs	-3.91320900	-0.12171000	-0.11708100
